# A review on the degradation of acetaminophen by advanced oxidation process: pathway, by-products, biotoxicity, and density functional theory calculation†

**DOI:** 10.1039/d2ra02469a

**Published:** 2022-06-22

**Authors:** Mohammad Qutob, Mahmoud A. Hussein, Khalid A. Alamry, Mohd Rafatullah

**Affiliations:** Division of Environmental Technology, School of Industrial Technology, Universiti Sains Malaysia 11800 Penang Malaysia mrafatullah@usm.my; Chemistry Department, Faculty of Science, King Abdulaziz University P.O. Box 80203 Jeddah 21589 Saudi Arabia mahussein74@yahoo.com maabdo@kau.edu.sa

## Abstract

Water scarcity and the accumulation of recalcitrance compounds into the environment are the main reasons behind the attraction of researchers to use advanced oxidation processes (AOPs). Many AOP systems have been used to treat acetaminophen (ACT) from an aqueous medium, which leads to generating different kinetics, mechanisms, and by-products. In this work, state-of-the-art studies on ACT by-products and their biotoxicity, as well as proposed degradation pathways, have been collected, organized, and summarized. In addition, the Fukui function was used for predicting the most reactive sites in the ACT molecule. The most frequently detected by-products in this review were hydroquinone, 1,4-benzoquinone, 4-aminophenol, acetamide, oxalic acid, formic acid, acetic acid, 1,2,4-trihydroxy benzene, and maleic acid. Both the experimental and prediction tests revealed that *N*-(3,4-dihydroxy phenyl) acetamide was mutagenic. Meanwhile, *N*-(2,4-dihydroxy phenyl) acetamide and malonic acid were only found to be mutagenic in the prediction test. The findings of the LC_50_ (96 h) test revealed that benzaldehyde is the most toxic ACT by-products and hydroquinone, *N*-(3,4-dihydroxyphenyl)formamide, 4-methylbenzene-1,2-diol, benzoquinone, 4-aminophenol, benzoic acid, 1,2,4-trihydroxybenzene, 4-nitrophenol, and 4-aminobenzene-1,2-diol considered harmful. The release of them into the environment without treatment may threaten the ecosystem. The degradation pathway based on the computational method was matched with the majority of ACT proposed pathways and with the most frequent ACT by-products. This study may contribute to enhance the degradation of ACT by AOP systems.

## Abstract 2

Researchers use advanced oxidation processes because of water scarcity and environmental recalcitrance. State-of-the-art studies on ACT by-products and their biotoxicity, proposed degradation pathways, have been collected, organized, and summarized.

## Graphical abstract

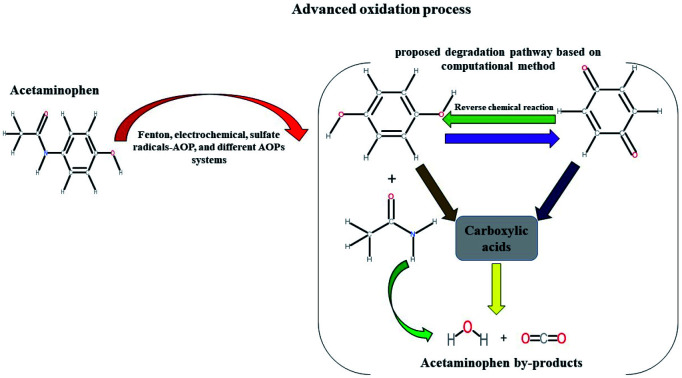

## 1. Introduction

Nowadays, pharmaceutical compounds have piqued the interest of environmentalists due to the rising demand for pharmaceutical compounds, which means a continuous release of them into the environment and little understanding of their effects and their by-products on human health and the environment.^1^ Pharmaceuticals compounds can flow to the environment from many sources like wastewater treatment plants (WWTPs), cure factories, domestic sewage, medical and research centers (unused, expired, and residual), animal husbandries, and landfills. Pharmaceuticals compounds have been detected in the surface water, groundwater, hospital effluent.^2^ The low-efficiency of wastewater treatment leads to releases the of pharmaceuticals into the water bodies. It has been observed that around 90% of pharmaceutical compounds that are excreted from the human body ending up in the aquatic ecosystem with passing time, these pharmaceuticals and their by-products accumulate in the fish, which is due to declining in fish fertility and cytotoxicity.^3^

Acetaminophen (ACT) or paracetamol (C_8_H_9_NO_2_, MW = 151.163, DrugBank Accession Number: DB00316) is one of the most popular pain killers use without a prescription for the relief of headache, backache, and rheumatic pains.^4,5^ It has been reported that around 6% of adults in the US consume more than 4 g per day, and more than 30 000 patients are hospitalized for ACT toxicity, which reflects the large consumption of ACT in the US.^6^ The researcher estimated the global production of ACT around 100 tons per year.^7^ This mass production of ACT increases the leakage opportunity into the environment, which is increases the threats of ACT and its by-products on the ecosystems.

In addition to pollutants accumulation into the environment, water scarcity is one of the main economic, social, and environmental problems in the 21st century. Thus, back to many reasons like increase the population, environmental change, and industrialization.^8^ To fulfill the rise in water demand and to avoid any further accumulation of contaminants into the environment, the researchers have proposed many water treatment approaches. These approaches are classified into three major classes (i) chemical treatment (ii) biological treatment (iii) physical treatment. Among them, advanced oxidation processes have gained attention to their ability to degrade high recalcitrance compounds.

Advanced oxidation process (AOP) is a chemical process based on activation of some molecules resulted in producing high electrophilic species or superoxide agents capable decomposing complex and highly recalcitrance pollutants. Many AOP techniques have been applied to oxidize ACT from an aqueous medium such as photocatalytic (*via* visible light or ultraviolet), ultrasound, Fenton, photo Fenton, photo-electro Fenton, AOP-based on nanomaterials, ozonation, thermal activation, and electro activation.^9–17^ The chemical eqn (1)–(6) are an example of the formation of the radicals when Fenton and iron/PS systems applied:

1

<svg xmlns="http://www.w3.org/2000/svg" version="1.0" width="23.636364pt" height="16.000000pt" viewBox="0 0 23.636364 16.000000" preserveAspectRatio="xMidYMid meet"><metadata>
Created by potrace 1.16, written by Peter Selinger 2001-2019
</metadata><g transform="translate(1.000000,15.000000) scale(0.015909,-0.015909)" fill="currentColor" stroke="none"><path d="M80 600 l0 -40 600 0 600 0 0 40 0 40 -600 0 -600 0 0 -40z M80 440 l0 -40 600 0 600 0 0 40 0 40 -600 0 -600 0 0 -40z M80 280 l0 -40 600 0 600 0 0 40 0 40 -600 0 -600 0 0 -40z"/></g></svg>

Fe(ii) + S_2_O_8_^2−^ → Fe(iii) + SO_4_˙^−^

2 Fe(ii) + H_2_O_2_ → Fe(iii)+ ˙OH

3 SO_4_˙^−^+ HO^−^ → SO_4_^2−^ + ˙OH

4 ˙OH + S_2_O_8_^2−^ → HO^−^ + S_2_O_8_˙^−^

5
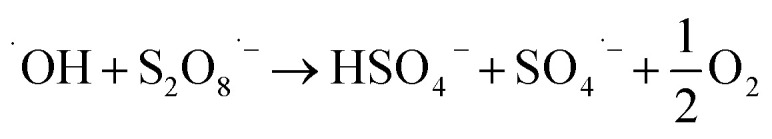

6 SO_4_˙^−^ + S_2_O_8_^2−^ → SO_4_^2−^ + S_2_O_8_˙^−^

In our previous work, we mentioned the influence of different parameters, degradation mechanism, degradation efficiency, and catalyst reusability for AOP systems that used to degrade ACT from an aqueous medium.^18^ In this review, we are going to collect, organize, and summarize the scattered information related to ACT proposed pathways, by-products, and their biotoxicity. This study also used a computational method to anticipate the ACT degradation pathway.

## 2. ACT degradation pathways

In AOP systems, many different treatment techniques have been applied to remove persistent organic pollutants from an aqueous medium, which generates several kinetics reactions and by-products. These by-products could be the same or different in types or concentrations. Since most remediation technologies are based on the application of appropriate degradation pathways, so, it is necessary to identify the degradation pathway of the target pollutant. There are many benefits to the determination of the degradation pathway like control the effectiveness of remediation system, the influence of degradation on analytical results can be eliminated, and the knowledge of degradation pathways for particular compounds can facilitate the assessment of environmental pollution based on the presence of degradation products. In addition, the identification of the degradation pathway is useful for the future development of a reaction mechanism and a kinetic model.^19^ Many studies have proposed degradation pathways of ACT based on the identification of the by-products during and after the chemical reaction. Table 1 represents the most frequent by-product molecules that proposed to build ACT degradation pathways.

**Table 1:** List of main proposed by-products to build ACT degradation pathways

Product number	Chemical formula and molecular weight	Chemical structure	Product number	Chemical formula and molecular weight	Chemical structure
P1	C_6_H_5_Cl_2_NO, *m*/*z*: 180	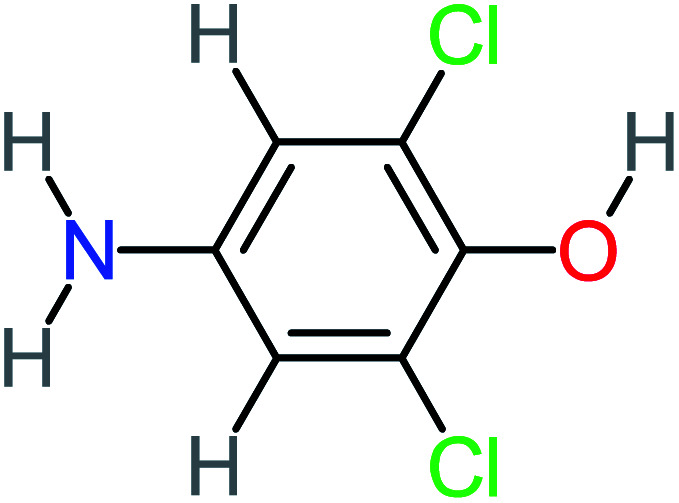	P31	C_2_H_2_O_4_, *m*/*z*: 90	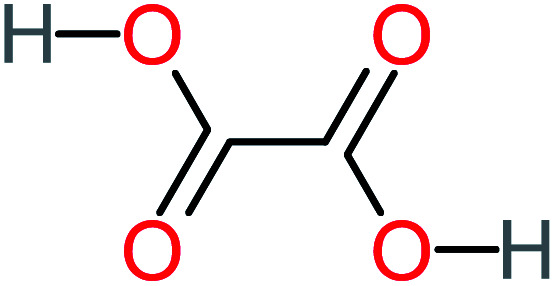
P2	C_8_H_11_NO_3_, *m*/*z*: 169	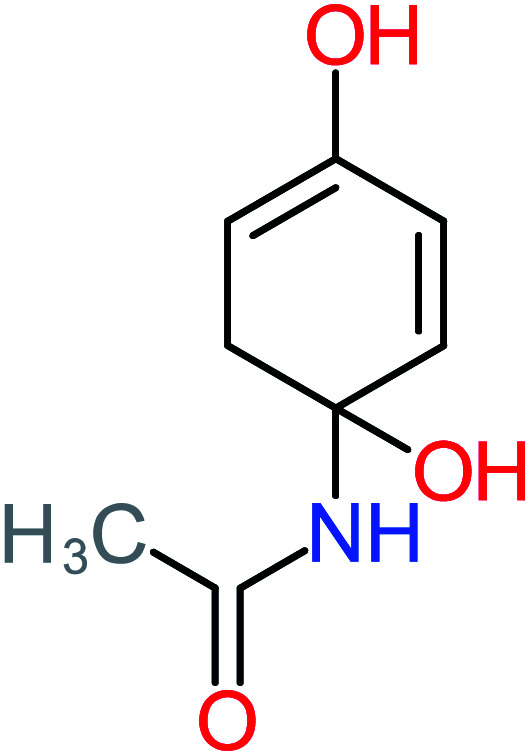	P32	C_4_H_8_O_2_, *m*/*z*: 88	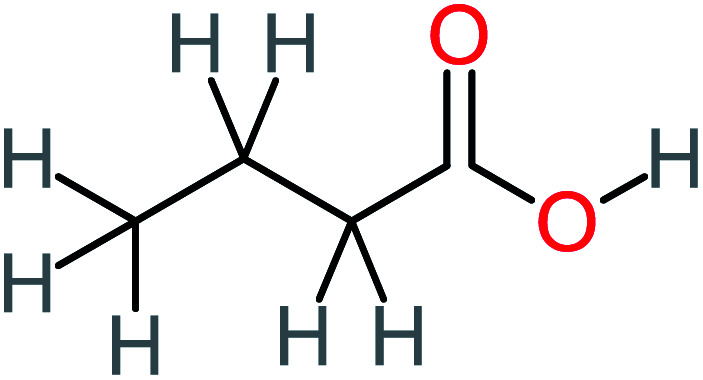
P3	C_8_H_9_NO_3_, *m*/*z*: 167	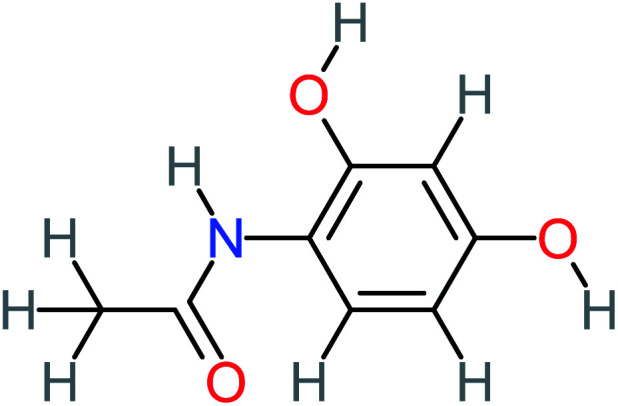	P33	C_4_H_4_O_4_, *m*/*z*: 116	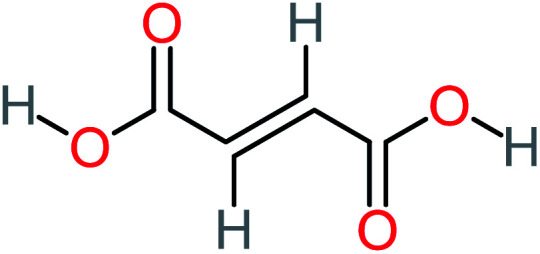
P4	C_6_H_6_O_2_, *m*/*z*: 110	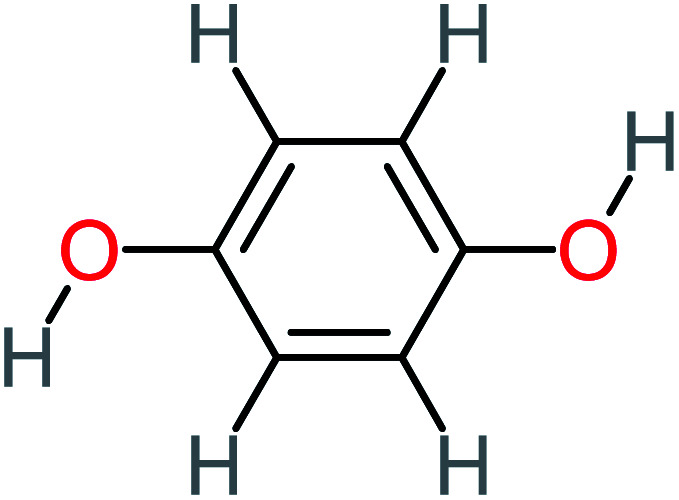	P34	C_4_H_6_O_6_, *m*/*z*: 148	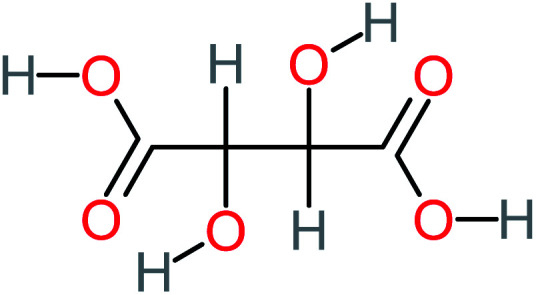
P5	C_2_H_5_NO, *m*/*z*: 59	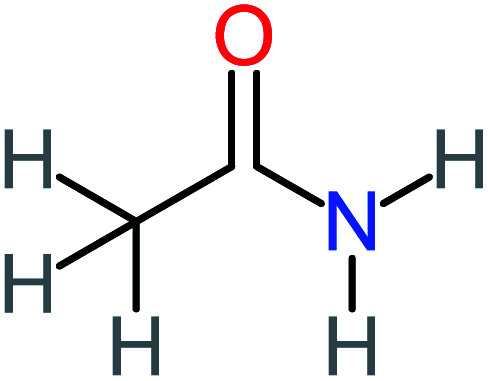	P35	C_4_H_6_O_5_, *m*/*z*: 134	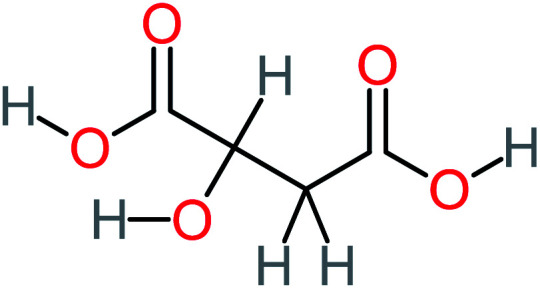
P6	C_6_H_7_NO, *m*/*z*: 109	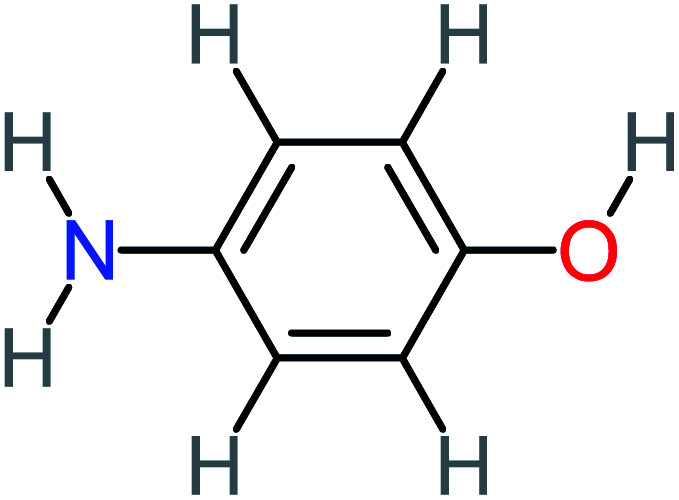	P36	C_2_H_4_O_2_, *m*/*z*: 60	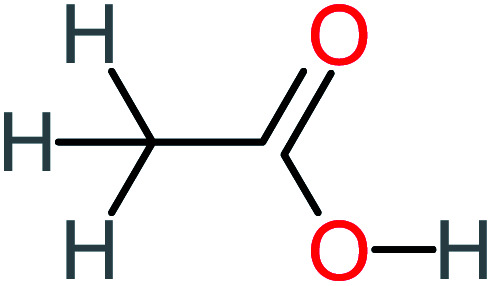
P7	C_8_H_9_NO_3_, *m*/*z*: 167	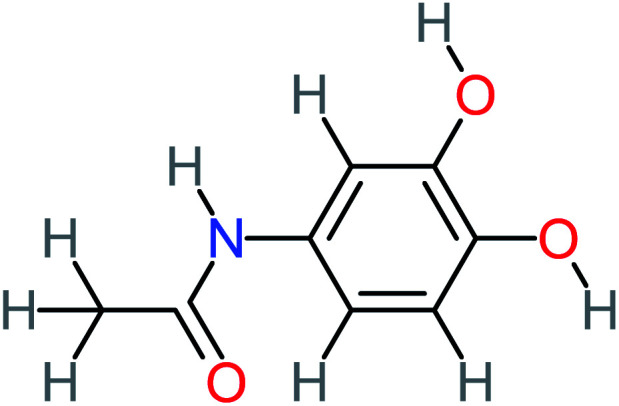	P37	NO_3_^−^, *m*/*z*: 62	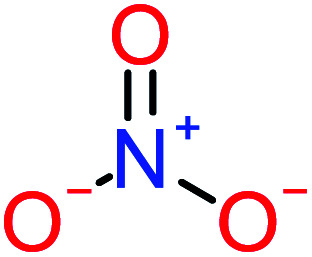
P8	C_6_H_4_O_2_(*m*/*z*: 108)	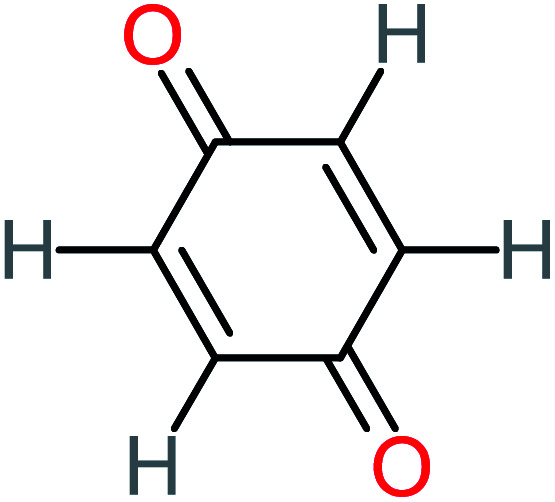	P38	CH_2_O_2_, *m*/*z*: 46	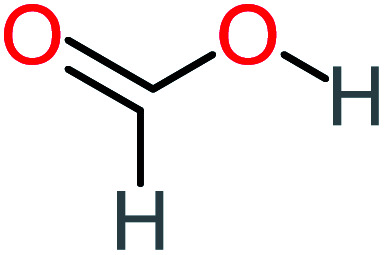
P9	C_8_H_11_NO_4_, *m*/*z*: 185	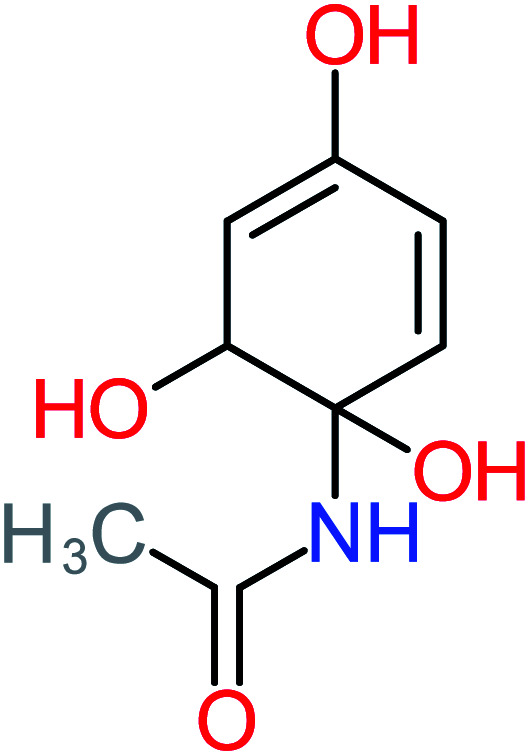	P39	C_2_H_7_N, *m*/*z*: 45	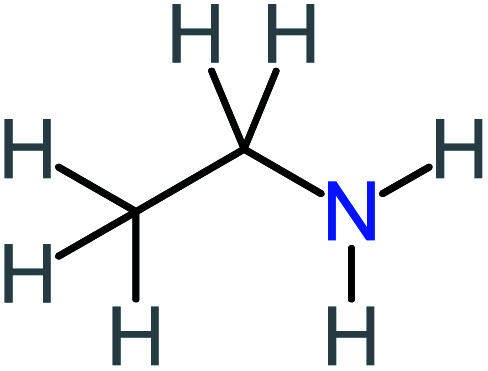
P10	C_6_H_6_ClNO, *m*/*z*: 145	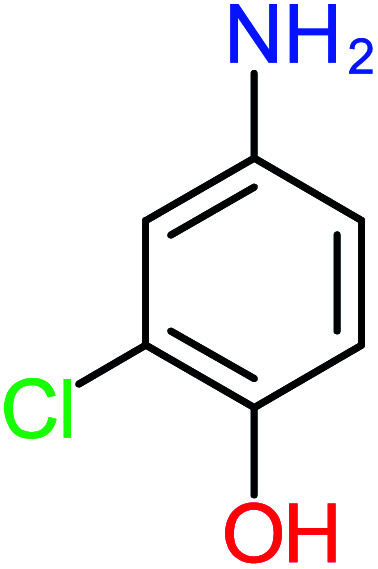	P40	C_8_H_9_NO_2_, *m*/*z*: 151	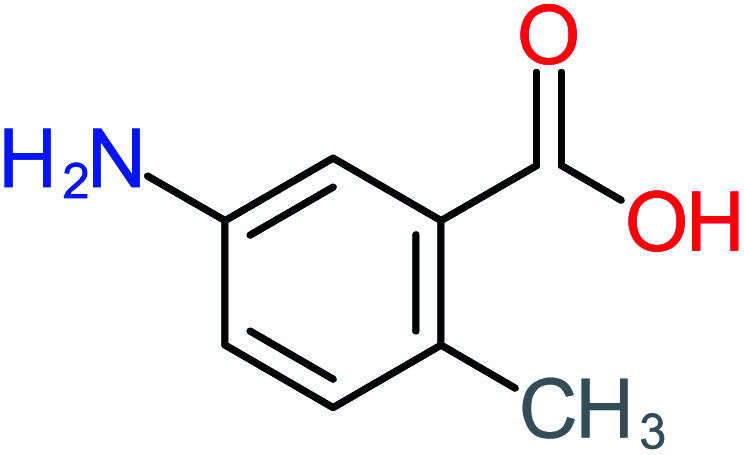
P11	C_6_H_6_O_3_, *m*/*z*: 126	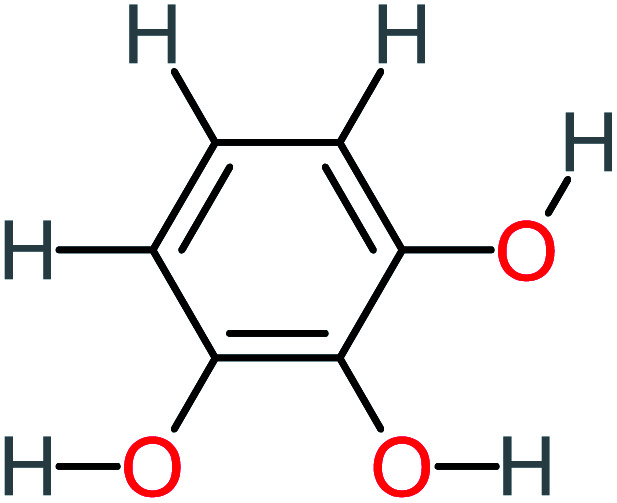	P41	C_7_H_8_O_2_, *m*/*z*: 124	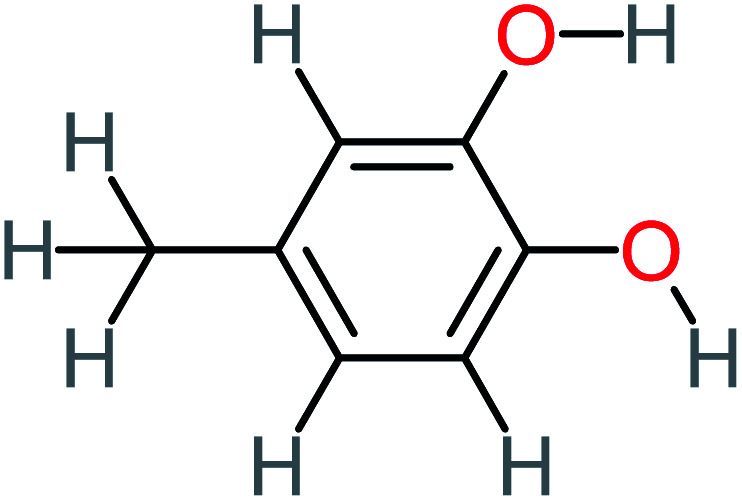
P12	C_6_H_5_NO_3_, *m*/*z*: 139	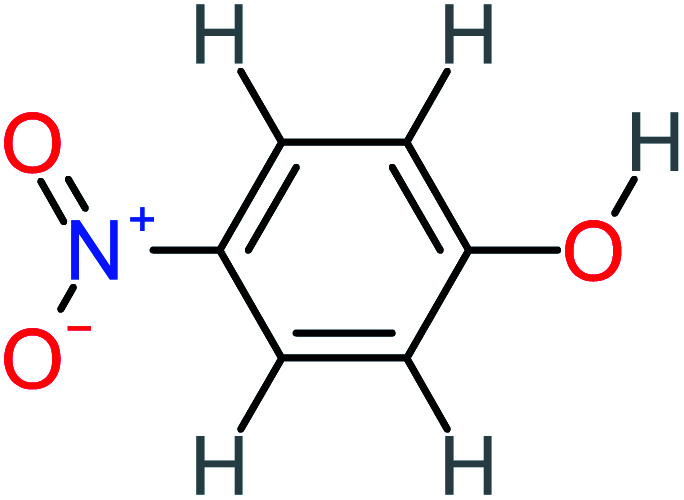	P42	C_6_H_4_N_2_O_5_, *m*/*z*: 184	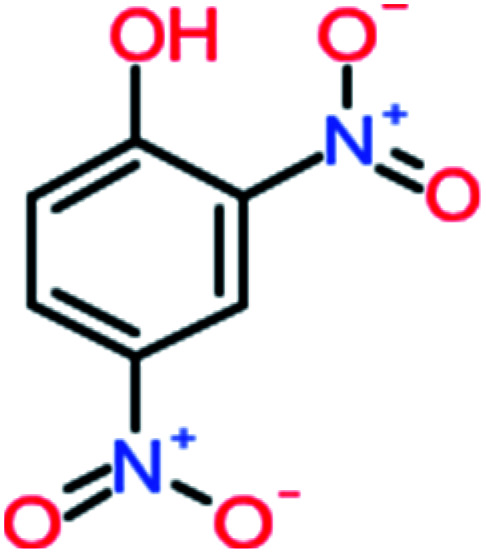
P13	C_16_H_16_N_2_O_4_, *m*/*z*: 301	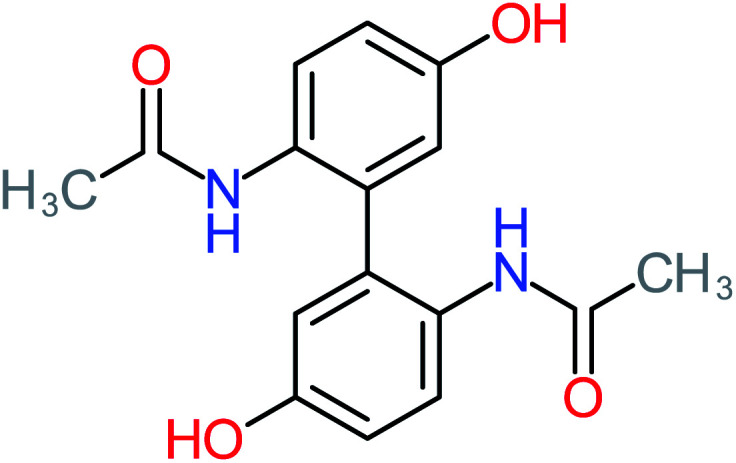	P43	C_5_H_11_NO, *m*/*z*: 101	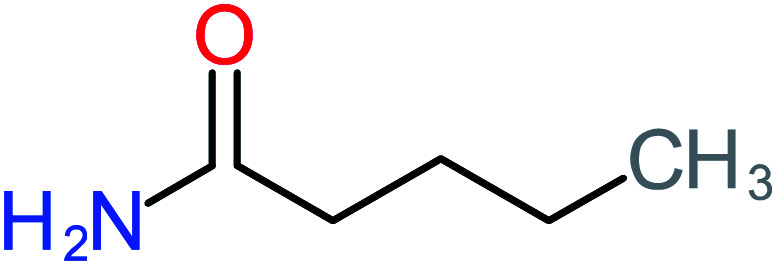
P14	C_5_H_7_NO_3_, *m*/*z*: 129	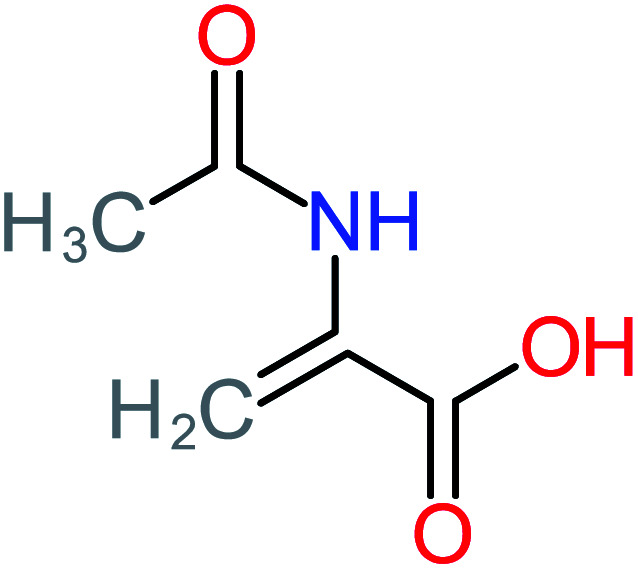	P44	C_7_H_5_ClO_2_, *m*/*z*: 157	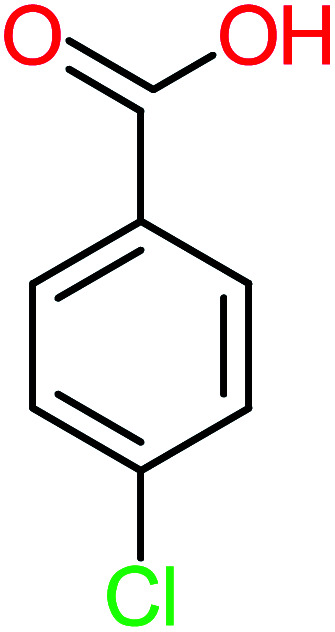
P15	C_8_H_7_NO_2_, *m*/*z*: 149	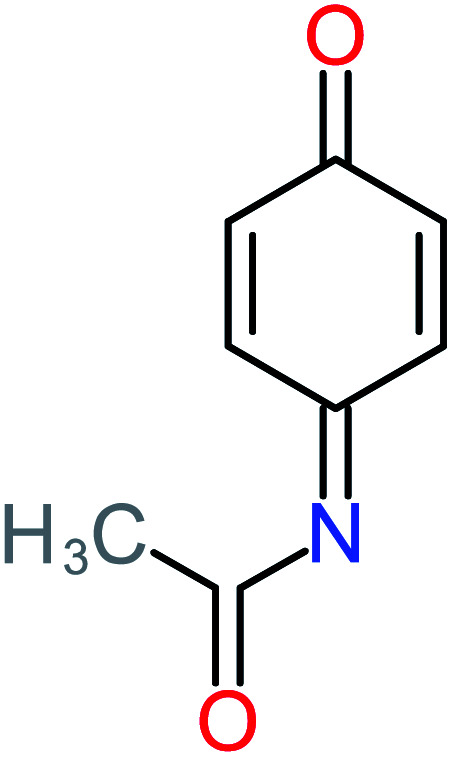	P45	C_6_H_6_O_2_, *m*/*z*: 110	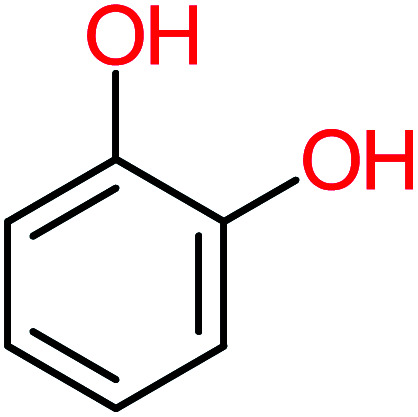
P16	C_6_H_6_O, *m*/*z*: 94	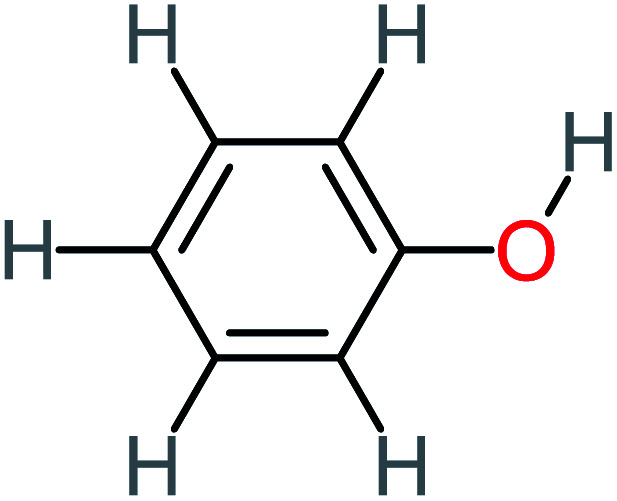	P46	C_7_H_8_O_2_, *m*/*z*: 124	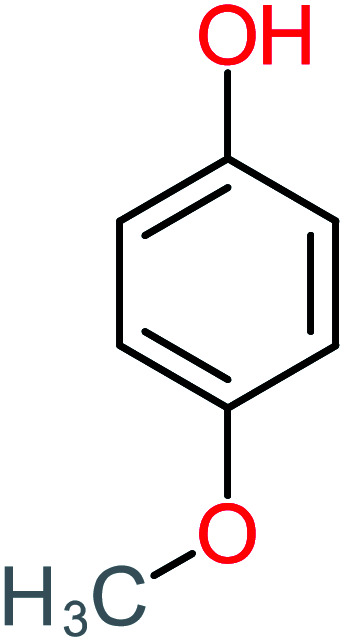
P17	C_8_H_9_NO_2_, *m*/*z*: 151	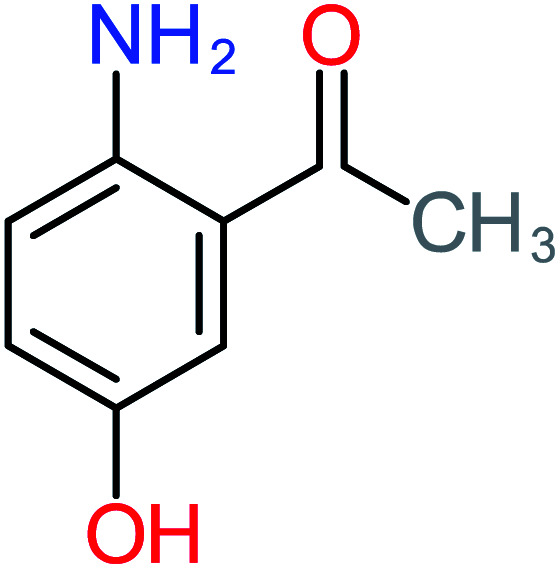	P47	C_4_H_9_NO_2_, *m*/*z*: 103	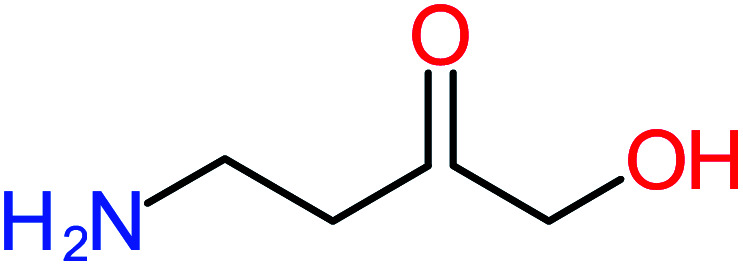
P18	C_7_H_7_NO_3_, *m*/*z*: 153	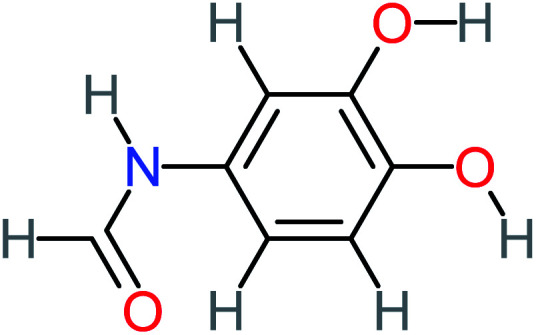	P48	CH_3_NO_2_, *m*/*z*: 61	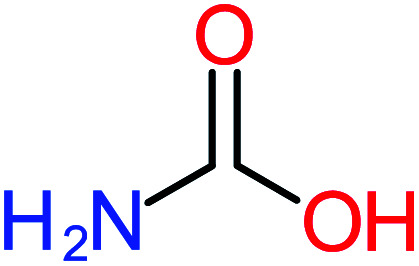
P19	C_2_H_3_NO_3_, *m*/*z*: 89	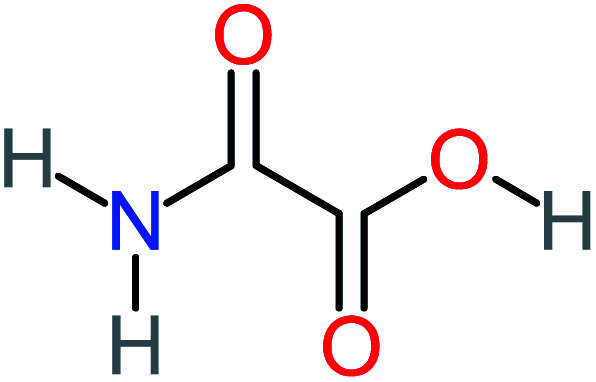	P49	C_8_H_9_NO_3_, *m*/*z*: 167	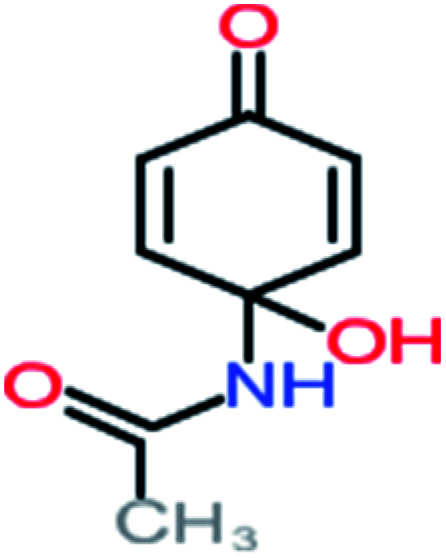
P20	C_8_H_8_O_2_, *m*/*z*: 136	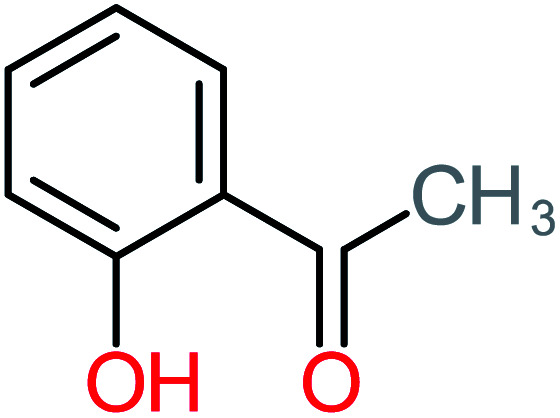	P50	C_4_H_11_N, *m*/*z*: 73	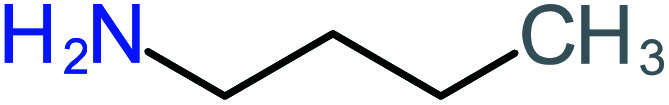
P21	C_7_H_6_O_2_, *m*/*z*: 122	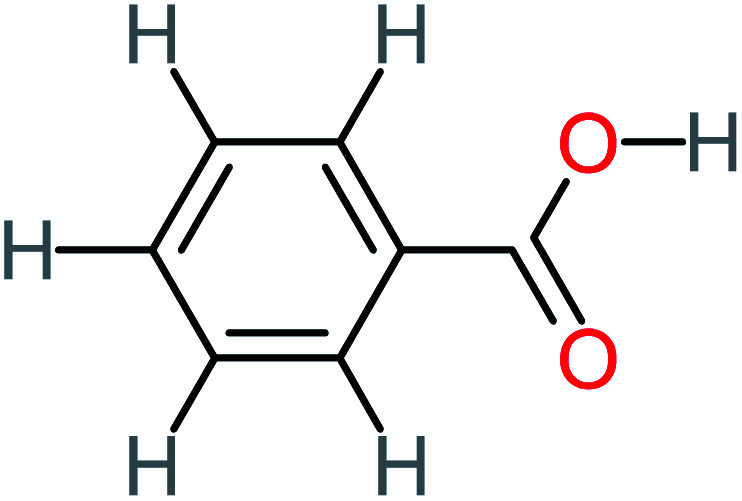	P51	C_7_H_9_NO, *m*/*z*: 123	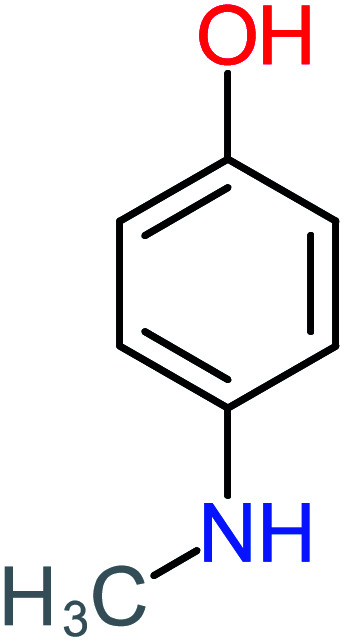
P22	C_7_H_8_O_2_, *m*/*z*: 124	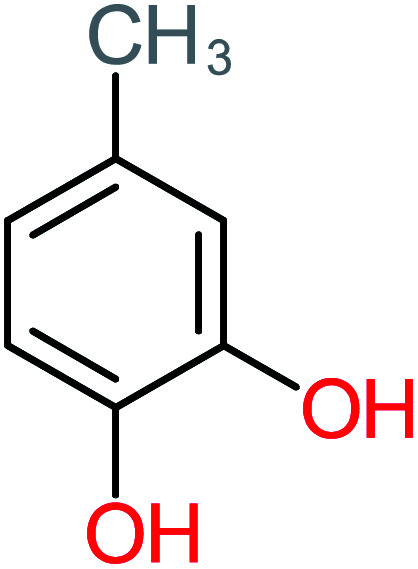	P52	C_6_H_8_NO, *m*/*z*: 110	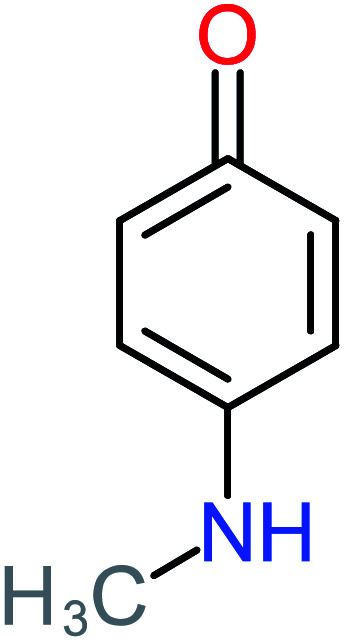
P23	NH_4_^+^, *m*/*z*: 18	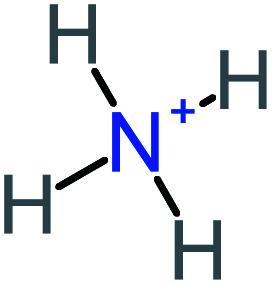	P53	C_4_H_6_O_4_, *m*/*z*: 118	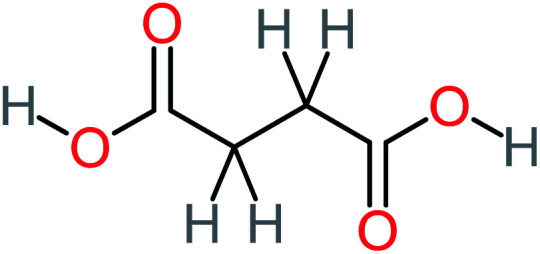
P24	C_8_H_7_Cl_2_NO_2_, *m*/*z*: 220	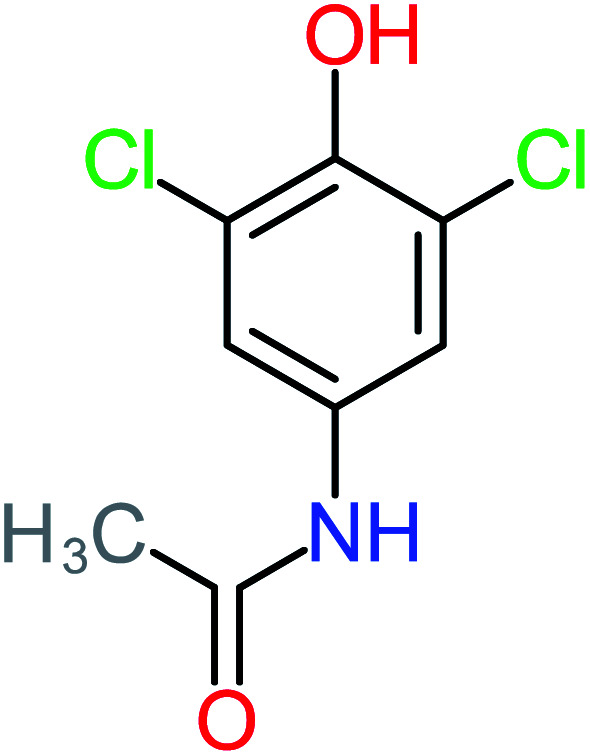	P54	C_8_H_9_NO_5_, *m*/*z*: 200	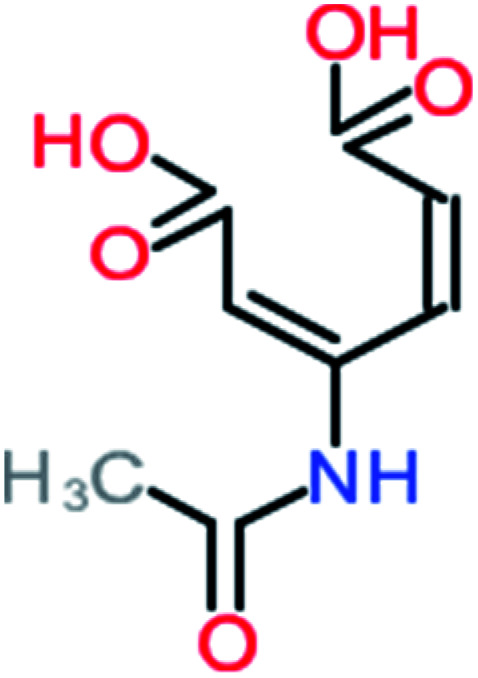
P25	C_4_H_4_O_4_, *m*/*z*: 116	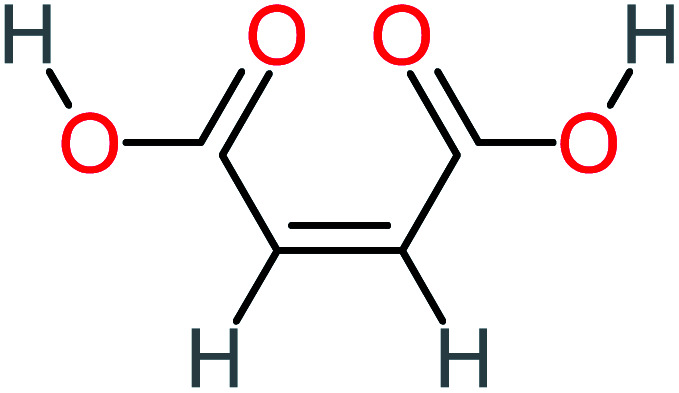	P55	C_6_H_6_O_4_, *m*/*z*: 142	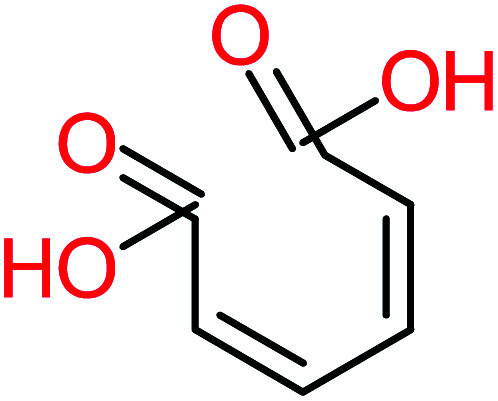
P26	C_2_H_4_O_3_, *m*/*z*: 76	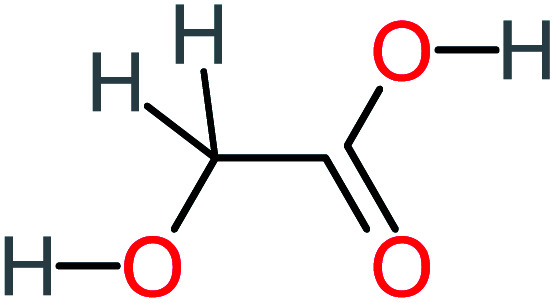	P56	C_6_H_7_NO_2_, *m*/*z*: 111	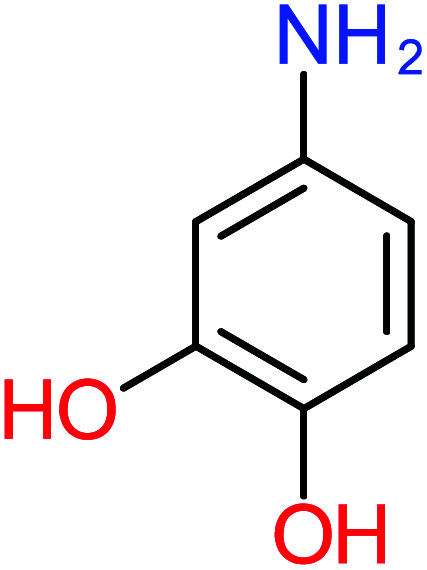
P27	C_7_H_7_NO_3_, *m*/*z*: 153	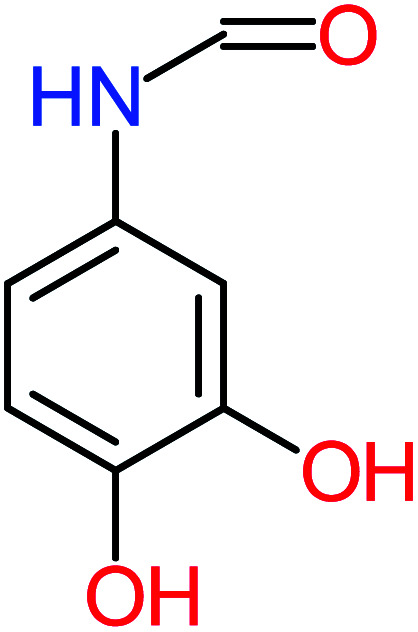	P57	C_7_H_16_O, *m*/*z*: 116	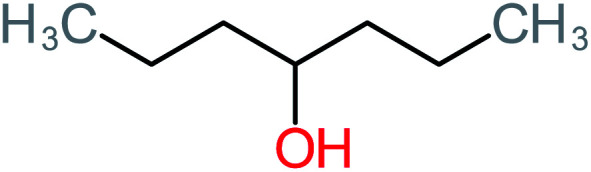
P28	C_3_H_4_O_4_, *m*/*z*: 104	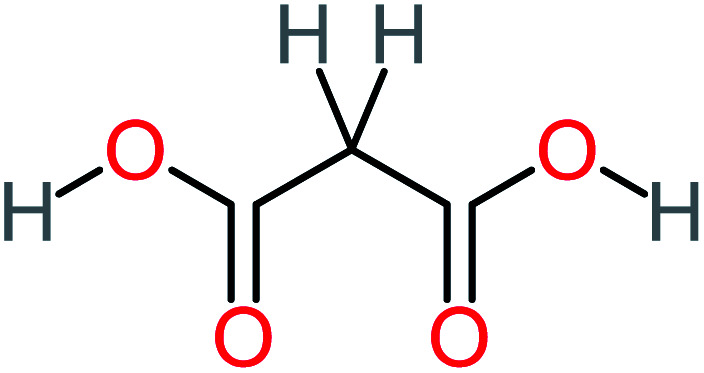	P58	C_6_H_12_O, *m*/*z*: 100	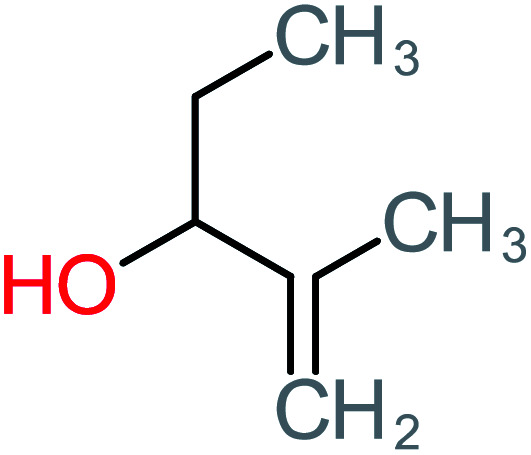
P29	C_7_H_6_O, *m*/*z*: 106	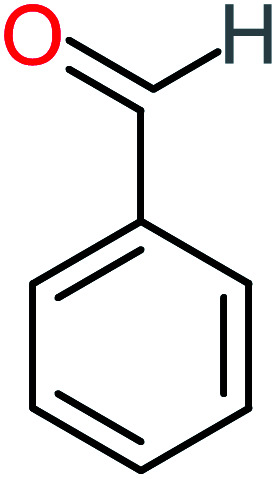	P59	C_3_H_6_O_3_, *m*/*z*: 90	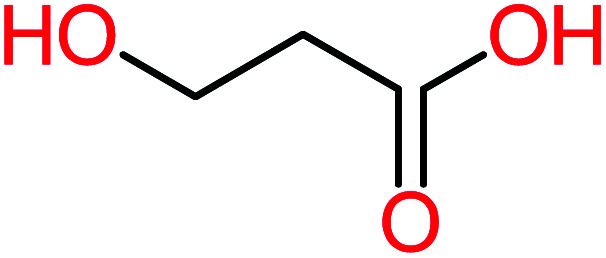
P30	C_6_H_5_ClO_2_, *m*/*z*: 145	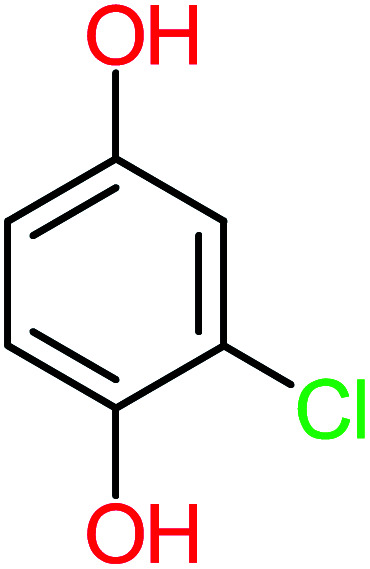			

According to the literature, we can classify the majority of the proposed ACT degradation pathways into three (i) coupling, which is the combination of phenoxyl radical and ACT to form ACT dimer P13, further oxidation of ACT dimer produces carboxylic acid.^20–23^ (ii) Direct cleavages of the ACT ring leading to form P54 then P55 → carboxylic acid → CO_2_ + H_2_O.^24–26^ (iii) Hydroxylation is the most dominant proposed pathway of ACT. The radical may attack *para*, *ortho*, or *meta* positions in the ACT ring leads to form *N*-(3,4-dihydroxyphenyl) acetamide P7 or *N*-(2,4-dihydroxyphenyl)acetamide P3. Further oxidation of P7 and P3 thus leads to produce hydroquinone P4, 1,4-benzoquinone 8, and acetamide P5, further oxidation of P4, P8, and P5 leads to forms carboxylic acid. Complete mineralization of carboxylic acid leading to form CO_2_ + H_2_O. In addition, if the radical attacks the N atom in the ACT molecule, thus due to form 4-aminophenol P6 then 4-nitrophenol P12, further oxidation of P12 leading to form P4 and P8. If the radical attacks *para* position in the ACT ring, this leading to produce P4 and P5, further oxidation of P4 produce P8 more oxidation of P8 leading to form carboxylic acid then CO_2_ and H_2_O, also further oxidation of p5 may producing acetic acid P36, formic acid P38, ammonium P23. It should be mentioned that hydroxylation pathways were the most abundant proposed degradation of the ACT pathway, especially hydroquinone and 4-aminophenol pathways. Skoumal *et al.*^5^ examined O_3_/Fe^2+^ + Cu^2+^/UV system to oxidize ACT. They proposed the degradation pathway based on the detected by-products. The radicals may target C2 in the ACT molecule, resulting in 2-hydroxyl-4-(4-acetyl)aminophenol production. Furthermore, the radicals may target C4, resulting in hydroquinone and acetamide. Further degradation of 2-hydroxyl-4-(4-acetyl)aminophenol generated glyoxylic acid and ketomalonic acid. The oxidation of hydroquinone leads to the formation of 1,4-benzoquinone, then the ring cleavages produced carboxylic acids and that, the acids were converted into CO_2_ and H_2_O. Ganiyu *et al.*^27^ applied the electrochemical system for ACT decomposition from an aqueous medium. In this study, three degradation pathways were proposed. (i) *N*-Dealkylation process for the ACT, which generated hydroquinone and acetamide. Further oxidation of hydroquinone giving carboxylic acids and ammonium then CO_2_ and H_2_O. (ii) The radicals attacked peptide bond giving 4-aminophenol, the hydroxylation of 4-aminophenol leading to formation hydroquinone then benzene ring cleavage giving carboxylic acids. (iii) Hydroxylation of ACT molecules produced 2-hydroxyl-4-(4-acetyl)aminophenol, further oxidation of 2-hydroxyl-4-(4-acetyl)aminophenol leading to formation hydroquinone. Gao *et al.*^28^ proposed three degradation pathways of ACT, pathway (i) was formed when the aromatic ring of ACT was hydroxylated, resulting in the creation of *N*-(3,4-dihydroxyphenyl) acetamide, then the aromatic ring of *N*-(3,4-dihydroxyphenyl) acetamide is cleaved, resulting in the creation of a ring opening product. Pathway (ii) began with the attack of the ˙OH on the *para* position of the phenolic functional group, resulting in the synthesis of hydroquinone, which was then oxidized to generate 1,2,4-trihydroxybenzene. Pathway III initiated the attack of ˙OH on the acetyl-amino group, leading to the formation of 4-aminophenol, which was then oxidized to 4-nitrophenol. Fan *et al.*^29^ implemented the Ag/AgCl@ZIF-8/visible light system to degrade ACT. They mentioned that hydroxylation and photolysis were the first steps of ACT oxidation. The radical attacked C1 and C4 parallelly, which led to the formation of 1,4-benzoquinone. Further oxidation of 1,4-benzoquinone leads to producing carboxylic acids then CO_2_ and H_2_O. Moreover, De Luna *et al.*^30^ studied electrochemical system for ACT degradation. They proposed that ˙OH prefers to attack *para* position in the aromatic ring in ACT, which leads to produce hydroquinone and acetamide. Further oxidation of hydroquinone giving benzaldehyde then turned into benzoic acid leading to ring cleavages and giving alcohols and small carboxylic acids. Table 2 represents the proposed oxidation pathways of ACT by different AOP systems and their active oxidation agents.

**Table 2:** Proposed oxidation pathways of ACT for more than 40 studies for different AOP systems

Systems	Proposed pathways	Active radicals	References
O_3_/Fe^2+^ + Cu^2+^/UV	ACT → P4 → P8 → carboxylic acids → H_2_O + CO_2_	˙OH	5
	P5 → P36, P38, or P23		
Fe^2+^/PS	(1) ACT → P6 → P4 → carboxylic acids → H_2_O + CO_2_	SO_4_˙^−^ and ˙OH	15
	(2) ACT → P7 → P22 + P5		
	P22 → carboxylic acids → H_2_O + CO_2_		
	P7 → P21 + P5		
	P21 → carboxylic acids → H_2_O + CO_2_		
	P5 → P36, P38, or P23		
MgO/O_3_	(1) ACT → P7 → P5 + P11	˙OH	31
	P11 → P25 → P28 → P38		
	P5 → P36, P38, or P23		
	(2) ACT → P2 → P5 + P4		
	P4 → P11 → P25 → P28 → P38		
	P5 → P36, P38, or P23		
Photocatalytic degradation	(1) ACT → P2 → P5 + P4	˙OH	32
	P4 → carboxylic acid → H_2_O + CO_2_		
	(2) ACT → P49 → P4 → P5 → carboxylic acid → H_2_O + CO_2_		
Iron–copper/persulfate/PS	(1) ACT → P6 → P4 + P5	SO_4_˙^−^ and ˙OH	33
	P4 → carboxylic acid → H_2_O + CO_2_		
	(2) ACT → P7 → P5 + P11		
	P11 → carboxylic acid → H_2_O + CO_2_		
TiO_2_/Fe_2_O_3_ core–shell nanostructure	(1) ACT → P3 → P11 → carboxylic acid → H_2_O + CO_2_	˙OH	34
	(2) ACT → P4 → P11 → carboxylic acid → H_2_O + CO_2_		
	(3) ACT → P7 → P11 → carboxylic acid → H_2_O + CO_2_		
Electro-Fenton and photoelectro-Fenton	ACT → P2 → P4 + P5	˙OH	30
	P4 → P29 → P21 → carboxylic acid → H_2_O + CO_2_		
	P5 → P39 → P23 → P37		
Catalytic wet peroxide oxidation (CWPO)	(1) ACT → P8 → P21 or P29 → P28, P36, P38, or P3 → H_2_O + CO_2_	˙OH	35
Solar light/Ag-g-C_3_N_4_/O_3_	ACT → P7 → P54 → P55 → carboxylic acids → H_2_O + CO_2_	h^+^ and ˙OH	24
La-doped	(1) ACT → P7 → P40 → P21 → H_2_O + CO_2_	˙OH	36
ZnO photocatalyst	(2) ACT → P9 → P8 + P5		
	P8 → P4 → P19 → H_2_O + CO_2_		
	P5 → P23		
Ag/AGCl@ZIF8/visible light	(1) ACT → P8 → carboxylic acid → H_2_O + CO_2_	O_2_˙^−^	29
	(2) ACT → P16 + P5		
	P16 → carboxylic acid → H_2_O + CO_2_		
Peracetic acid/UVC-LED/Fe(ii)	(1) ACT → P4 + P5	˙OH	37
	(2) ACT → P56 → P4 → carboxylic acid → H_2_O + CO_2_		
	(3) ACT → P12 or P4 → carboxylic acid → H_2_O + CO_2_		
	(4) ACT → P7 → P56 → carboxylic acid → H_2_O + CO_2_		
ZVAl/H^+^/air system	ACT → P2 → P4 + P5	˙OH	38
	P4 → carboxylic acids → H_2_O + CO_2_		
	P5 → P36, P38, or P23		
CS–Fe/PS	(1) ACT → P16 + P5	˙OH and SO_4_˙^−^	39
	P16 → P4 → P8 → carboxylic acid → H_2_O + CO_2_		
	P5 → P39 → P23		
	(2) ACT → P6 + P36		
	P39 → P43 + P39		
	P43 → carboxylic acid → H_2_O + CO_2_		
Cobalt-impregnated biochar/PMS	(1) ACT → P7 → P56 → P6 or P11	˙OH and SO_4_˙^−^	40
	(2) ACT → P56 → P6 or P11		
	(3) ACT → P6 → P4 → carboxylic acid → H_2_O + CO_2_		
	P11 → P4 → carboxylic acid → H_2_O + CO_2_		
	P6 → P4 → carboxylic acid → H_2_O + CO_2_		
Heat/peroxymonosulfate system	ACT → P6 → P56 or P12	^1^O_2_ and ˙OH	41
	P12 → P25 or P28 → P36, P38, or P31 → CO_2_ + H_2_O		
	P56 → P31 → CO_2_ + H_2_O		
Ferrous ion/copper oxide O_2_	ACT → P2 → P4 → + P5	˙OH	42
	P4 → P3 → P38 or P36 → H_2_O + CO_2_		
Fenton process by plasma gliding arc discharge	(1) ACT → P4 → P8 → carboxylic acid → H_2_O + CO_2_	˙OH	43
	(2) ACT → P42 → P8 → carboxylic acid → H_2_O + CO_2_		
SnO_2_/O_3_	(1) ACT → P7 → carboxylic acid → H_2_O + CO_2_	˙OH	44
	(2) ACT → P4 + P5		
	P4 → carboxylic acid → H_2_O + CO_2_		
	P5 → P36 → P23		
OVPTCN/visible light	(1) ACT → P4 → P8 or P41 → P35 → H_2_O + CO_2_	˙OH	45
UV/H_2_O_2_	ACT → P11, P7, P8, or P4 → carboxylic acid → H_2_O + CO_2_	˙OH and halide radicals	46
UV-LED/NH_2_Cl and PS	(1) ACT → P4 or P6 → P8 or P11 → carboxylic acid → H_2_O + CO_2_	˙OH, Cl˙ and SO_4_˙^−^	47
	(2) ACT → P7 → P21 → P44 → carboxylic acid → H_2_O + CO_2_		
Photo Fenton-like oxidation process	(1) ACT → P13	O_2_˙^−^	21
	(2) ACT → P7 → P31 or P28 → P36 or P38 → H_2_O + CO_2_		
Photocatalytic degradation	(1) ACT → P13	O_2_˙^−^ and ˙OH	22
	(2) ACT → P50, P28, or P33 → P36 or P38 → H_2_O + CO_2_		
Photocatalytic degradation	ACT → P4 + P5	h^+^ and O_2_˙^−^	48
	P4 → P11 → P38 or P25 → CO_2_ + H_2_O		
	P5 → P37 + CO_2_ + H_2_O		
Photocatalytic degradation	ACT → P51 → P6 → P52 → P8 → P38 → P36 → CO_2_ + H_2_O	O_2_˙^−^, ^1^O_2_ and ˙OH	49
Photocatalytic	ACT → P6 → P16 → P4 → CO_2_ + H_2_O	˙OH	50
Electro-Fenton process	(1) ACT → P4 + P5	˙OH	51
	P4 → P8 or P11 → carboxylic acid → H_2_O + CO_2_		
	(2) ACT → P3 → P8 → carboxylic acid → H_2_O + CO_2_		
Electrochemical degradation	(1) ACT → P6 → P4	˙OH	27
	(2) ACT → P4+P5		
	P4 → P8 → carboxylic acid → H_2_O + CO_2_		
	P5 → P36, P38, or P23		
Electro-Fenton	(1) ACT → P8 → P21 or P29 → carboxylic acid → H_2_O + CO_2_	˙OH	52
	(2) ACT → P7 → P21 or P29 → carboxylic acid → H_2_O + CO_2_		
Electrocatalytic degradation	(1) ACT → P7 or P3 → P5 + P21	˙OH	53
	P21 → carboxylic acid → H_2_O + CO_2_		
	(2) ACT → P4 + P5		
	P4 → P11 or P8 → carboxylic acid → H_2_O + CO_2_		
	P5 → P23 → P37 + CO_2_ + H_2_O		
Electro-catalytic activation	ACT → P6 → P4 → P8 → P31 → CO_2_ + H_2_O	˙OH	54
Heterogeneous electro-Fenton process	ACT → P5 → P16	˙OH	55
	P16 → P57 or P58 → carboxylic acid → H_2_O + CO_2_		
Photo-Fenton	ACT → P4 + P5	˙OH	19
	P4 → P8 → carboxylic acid → H_2_O + CO_2_		
	P5 → P19 → P23 → CO_2_		
High active amorphous Co(OH)_2_/PMS	(1) ACT → P13	˙OH and SO_4_˙^−^	26
	(2) ACT → P6 → P4 + P5		
	P4 → P8 → P28 → CO_2_ + H_2_O		
	P5 → P36 or P38		
	(3) ACT → P7 → P56 or P54		
	P56 → P11 → P53 → CO_2_ + H_2_O		
	P54 → P55 → P53 → CO_2_ + H_2_O		
BaTiO_3_/TiO_2_ composite-assisted photocatalytic	ACT → P2 → P4 → P8 → carboxylic acid → H_2_O + CO_2_	˙OH	56
Fuel cell-Fenton system	ACT → P6 + P36	˙OH	57
	P6 → P12 → P25 → P35 or P28		
Electrochemical oxidation	ACT → P3 + P36	˙OH and SO_4_˙^−^	58
	P6 → P4 or P12 → P8 → carboxylic acid → H_2_O + CO_2_		
Photo-electrooxidation	ACT → P4 + P5	˙OH	59
	P4 → P8 → carboxylic acid → H_2_O + CO_2_		
Biotemplated copper oxide catalysts over graphene oxide for ACT removal	ACT → P4 → P8 → carboxylic acid → H_2_O + CO_2_	˙OH	60
Gas phase dielectric barrier discharge plasma combined with the titanium dioxide-reduced graphene oxide	ACT → P4 → P46 → carboxylic acid → H_2_O + CO_2_	˙OH	61
Photocatalytic degradation of acetaminophen	(1) ACT → P13	˙OH	62
	(2) ACT → P4 → P8 → carboxylic acid → H_2_O + CO_2_		
	(3) ACT → P7 → P11 → carboxylic acid → H_2_O + CO_2_		
	(4) ACT → P3 → P11 → carboxylic acid → H_2_O + CO_2_		
Degradation of acetaminophen by ferrate (vi)	ACT → P16 or P52 → P25 → P38 → P31 → H_2_O + CO_2_	Direct oxidation	63
Photocatalytic degradation of paracetamol	(1) ACT → P2 → P4 + P5	˙OH	64
	P4 → P8 → carboxylic acid → H_2_O + CO_2_		
	(2) ACT → P5 + P6 → P8 → carboxylic acid → H_2_O + CO_2_		

## 3. ACT degradation pathway based on computational method

In the treatment systems that are based on chemical oxidation, there are two major degradation mechanism pathways (1) non-radical pathway in this pathway, factors such as (irradiation, ultrasonic wave, electron transfer process, *etc.*) responsible for the degradation of the target pollutant, these factors can oxidize the pollutant spontaneously from any site, which increase the difficulty to predict the degradation pathway through a computational method, (2) radical pathway in this pathway, the radicals such as (˙OH, SO_4_˙^−^, and O_2_˙^−^) are responsible on the pollutant oxidation. In AOP systems, the radical pathway is mostly dominant and the radicals prefer to attack the highest occupied molecular orbital (HOMO) site on the target pollutant, which can predict the degradation pathway by computational method. Density functional theory (DFT) has been using to calculate the nucleophilic (*f*^+^), electrophilic (*f*^−^), and radical attack (*f*°) of each atom within the molecule.^65^ Fukui function *f*(*r*) is the best descriptor method for DFT.^66^ The following eqn (7)–(10) represents the Fukui functions.7
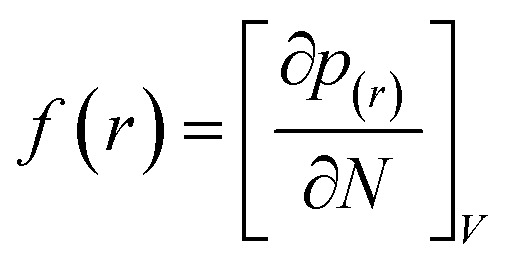

8 *f*^+^ = [*q*_*i*_(*N* + 1) − *q*_*i*_(*N*)]

9 *f*^−^ = [*q*_*i*_(*N*) − *q*_*i*_(*N* − 1)]

**Fig. 1:**
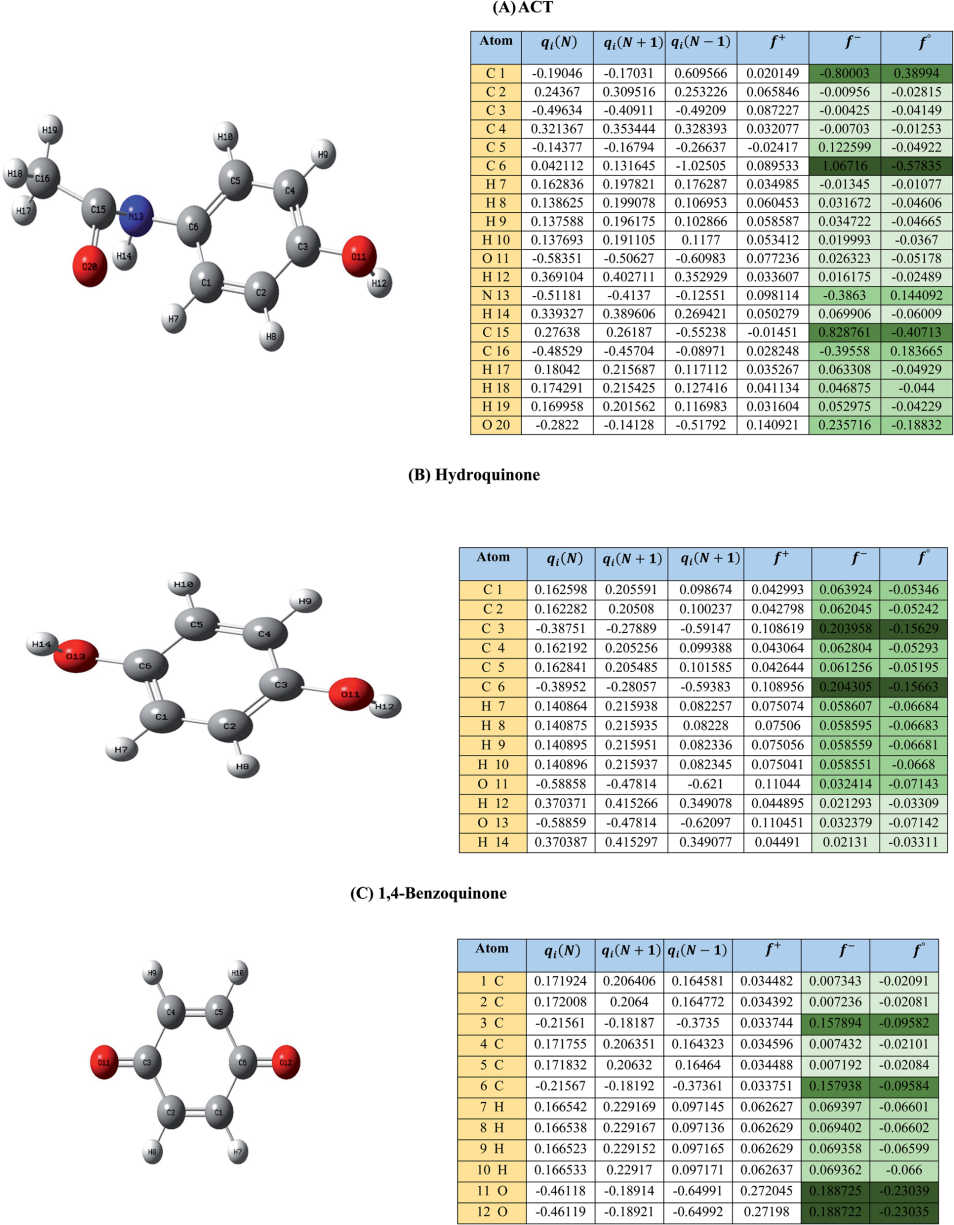
(A) Represent ACT molecule and its *f*^−^, *f*^+^, and *f*° values. (B) Hydroquinone, and (C) 1,4-benzoquinone.

10
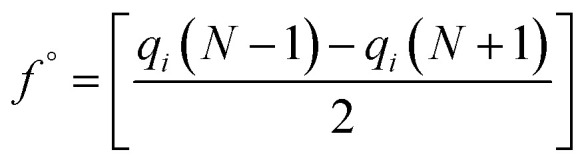
*p*(*r*) is the electron density at point (*r*) in the space, *qi* is the atomic charge, and *N* is the number of electrons. The previous studies which investigated the active sites of ACT by using DFT or frontier orbital theory did not provide enough information to build the degradation pathway of ACT.^25,26^ In this study, GaussView 6.0 and Gaussian 09 were used to execute the obtained data. Additionally, as a basis set, 6-31 G (d,p) and B3LYP (Becke's three parameters and Lee–Yang–Parr functional) were utilised.^67^Fig. 1 depicted the *f*^−^, *f*^+^, and *f*° values for each ACT, hydroquinone, and 1,4-benzoquinone and their chemical structure.

**Fig. 2:**
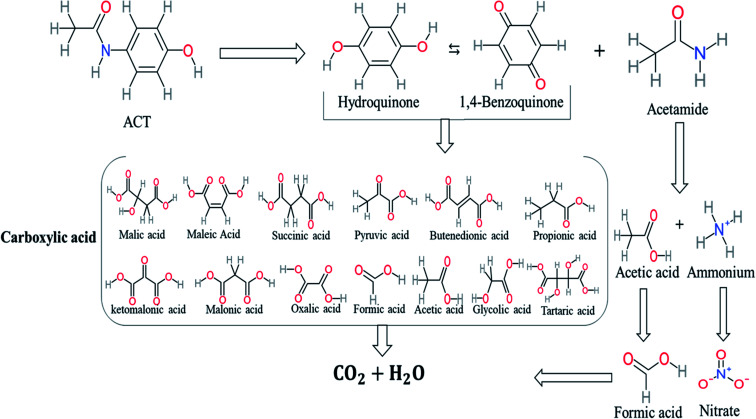
Illustrated the proposed degradation pathway based on computational method.

Based on the values illustrated in Fig. 1, the highest (*f*°) and (*f*^−^) values represent HOMO which is easier to lose an electron and readily attacked by electrophilic or oxidizing agents.^61,68^ This study revealed the highest value of (*f*^−^) was C6 (*f*^−^ = 1.06716) which means that the first attack of radical is C6 position. The radical attack on C6 results in the hydroxylation of the C6 position, resulting in the release of acetamide and the substitution of a hydroxyl group. Thus, leading to form hydroquinone and acetamide, the same results were obtained by ref. 69. According to Fig. 2, further degradation of acetamide leading to form acetic acid and ammonium, more oxidation of acetic acid and ammonium produce formic acid and nitrate, respectively. The highest (*f*^−^) value of hydroquinone were (*f*^−^ = 0.203958) and (*f*^−^ = 0.204305) for C3 and C6, respectively. In this case, there are three possible pathways (i) quick hydroxylation of C3 and C6 leading to formation 1,4-benzoquinone, (ii) if the radical attack C3 and C6 leading to the cleavage of the benzene ring, which is due to the form of small carboxylic acid such as glycolic acid, acetic acid, formic acid, pyruvic acid, oxalic acid, (iii) the values of (*f*^−^) for C6 was little bet higher than C3 which leading to ring cleavage from C6 position, leading to form carboxylic acids like malic acid, maleic acid, succinic acid, butenedionic acid, and tartaric acid. For pathway (i) further oxidation of 1,4-benzoquinone due to a reversible chemical reaction between hydroquinone and 1,4-benzoquinone. Since the highest values (*f*^−^) for 1,4-benzoquinone were (*f*^−^ = 0.188725) for O11 atom and (*f*^−^ = 0.188722) for O12 atom, in this case, the radical attack (O=C) bond for O11 and O12, resulting to reform of hydroquinone, this agreed with.^27,36,59,70^ For pathway (ii) further oxidation of the small carboxylic acid leading to completely mineralization and produce CO_2_ and H_2_O. Pathway (iii) more decomposition of carboxylic acid due to form small carboxylic acid such as glycolic acid, acetic acid, formic acid, pyruvic acid, oxalic acid, then convert to CO_2_ and H_2_O. Fig. 2 illustrate the degradation pathway of ACT based on the computational method. The predicted ACT pathway is matched with the majority of the proposed degradation pathways in the Table 2. In addition, the most frequent by-products of ACT that have been detected as hydroquinone, 1,4-benzoquinone, acetamide, formic acid, acetic acid, oxalic acid, and maleic acid, was predicted in this study by using computational method. Finally, computational chemistry assists the researchers in predicting the degradation pathway, especially for large organic molecules.

## 4. By-products of ACT

The specific objective of the chemical oxidation treatment is to mineralize the pollutant completely and convert them into CO_2_, NO_3_^−^, and H_2_O or convert them into harmless molecules. On the other hand, some AOP systems have partially mineralized the pollutant, which leads to producing by-products (also known as transformation products and intermediate products). These by-products could be threatened and toxic for the environment and public health more than the parent pollutant itself.^71^ The researcher illustrated the threaten of by-products that are released from WWTPs into the environment like an iceberg the pollutants themselves are just the tip of the iceberg while the by-products represent the majority of the iceberg that hidden underwater. As mentioned, many AOP systems have been applied to oxidize ACT from a liquid medium. Thus, leading to generate many of by-products. Many reduction-oxidation agents have been observed during the degradation of ACT, such as holes, photon, halide radicals, ozone, methyl radical, singlet oxygen, hydroxyl radical, sulfate radical, superoxide radical. These radicals may attack different sites of ACT, which leading to the formation of different and unique by-products. For example, Mashayekh-Salehi *et al.*,^31^ observed that the ozone molecule attacked the ACT molecule leading to the formation of 2-hydroxy-4-(*N*-acetyl)-aminophenol compounds. On the other hand, ozone molecules could not fully mineralized ACT because ozone does not have sufficient energy to do that. In addition, many studies observed that ACT dimer have been formed during ACT degradation. The mechanism behind the formation of ACT dimer was losing one electron, which changes the ACT molecule to cationic form (phenoxyl radical), then the self-combination of ACT with the neighbor phenoxyl radical leading to form ACT dimer.^20,32^ Methyl radicals and *N*-(3,4-dihydroxylphenyl)formamide were produced through the attack of ACT by OH° and methyl radicals attacked *N*-(3,4-dihydroxylphenyl)formamide and formed 4-methylbenzene-1,2-diol.^33^ Zhang *et al.*^72^ examined S-doped graphene/Pt/TiO_2_ to degrade ACT from an aqueous medium. They observed that chlorinated by-products such as 2-chlorohydroquinone and 4-chlorobenzene-1,2 diol were formed after attacking the ACT molecule by halides radicals. Abdel-Wahab *et al.*^34^ examined magnetic flower-like TiO_2_/Fe_2_O_3_ core–shell nanomaterials activated by irradiation. After the end of ACT degradation, the by-products were ACT, 4-acetamidocatechol, 4-acetamidoresorcinol, hydroquinone, 1,2,4-benzetiol, maleic acid, tartaric acid, malic acid, succinic acid, malonic acid, oxalic acid, oxamic acid, and acetamide. Kohantorabi *et al.*^73^ studied the oxidation of ACT by using Ag/ZnO@NiFe_3_O_4_ nanorods promoted by UVA/PMS. The by-products were hydroquinone, glycolic acid, 1,4-benzoquinone, and 3-hydroxypropanic acid. Zhang *et al.*^74^ were identified acetamide and benzoquinone. Then Benzoquinone was further oxidized to produce acetyl methyl carbinol, 2-pentanone, and methyl vinyl ketone as intermediates. Additionally, De Luna *et al.*^30^ applied photoelectro-Fenton using a double cathode electrochemical cell to decompose ACT from an aqueous medium. Acetic acid, formic acid, oxalic acid, malonic acid, hydroquinone, and amide were detected after 120 min of reaction. In addition, oxalic acid, formic acid, and acetic acid were the main transformation products when metal-loaded mesoporous for the catalytic wet peroxide oxidation of ACT.^35^ Fenton oxidation applied by De Luna *et al.*^75^ to degrade ACT. The by-products were hydroquinone, benzoic acid, benzaldehydes and some non-aromatic products like carboxylic acid, alcohols, ketones, and aldehydes. Yunfei Zhang *et al.*^76^ applied ferrous ion and copper oxide/O_2_ system to remove ACT from a liquid medium. The main by-products were hydroquinone, ammonium, formic acid, acetic acid, and oxalic acid. Furthermore, small carboxylic acid like formic acid, oxamic acid, and oxalic acid were detected when TiO_2_ nanotube activated by UV light was applied. Peng *et al.*^77^ used pyrite to activate persulfate and H_2_O_2_ for ACT degradation. In this system, the by-products were hydroquinone, acetamide, nitrate, and acetic acid. Platinum doped TiO_2_/photocatalytic systems were used to degrade ACT. After 60 min the transformation products were oxalic acid, acetic acid, and formic acid.^78^ Furthermore, Mashayekh-Salehi *et al.*^31^ applied MgO nanoparticles activated/O_3_ system to oxidize ACT from an aqueous medium. Malonic acid, succinic acid, malic acid, formic hydroxy acetic acid, acetamide, and nitrite were the major intermediate products in this system. Ling *et al.*^24^ carried out Ag–g-C_3_N_4_/O_3_ catalyzed by vis-UV light to oxidize ACT. Hydroquinone, di-hydroxyphenyl, and tri-hydroxyphenyl were the main by-products generated from this system. Thi & Lee^36^ implemented photocatalytic of 1%-La doped ZnO system to remove ACT from an aqueous atmosphere. Few by-products were produced in this system like hydroquinone, oxamic acid, acetic acid, butyric acid, and 2-amino-5-methyl benzoic acid. Moreover, G. Fan *et al.*^29^ pointed out that salicylaldehyde, acetamide, phenol, lactic acid, succinic acid, malic acid, and maleic acid were generated when Ag/AgCl@ZIF-8/visible light system was applied to oxidize ACT. In addition, hydroquinone, 1,4-benzoquinone, 4-methoxyphenol, 2-hexenic, and malic acid were monitored when oxygen vacancies and phosphorus coded black titania coated carbon nanotube composite activated by visible light was applied. Ghanbari *et al.*^37^ studied a synergistic peracetic acid/UVC-LED system to oxidize ACT. 4-Nitrophenol and hydroquinone were the transformation compounds in this system. H. Zhang *et al.*^38^ applied a zero valent aluminum-acid system to degrade ACT from a liquid medium. The main by-products were hydroquinone and anionic derivatives like acetate and nitrate. S. Wang *et al.*^15^ examined Fe^2+^/PS system to remove ACT. They detected hydroquinone, 1,4-benzoquinone, *N*-(3,4-dihydroxyphenyl)formamide, and 4-aminophenol, 4-methylbenzene-1,2-diol after 30 minutes of reaction. Pham *et al.*^79^ detected oxaloacetic acid and 4-nitrophenol were the major transformation products when Fe and N co-doped carbon nanotube system was applied. In this review, the by-products of 64 studies related to the oxidation of ACT from an aqueous medium by using different AOP systems were collected and summarized in the Table 3. This study revealed that hydroquinone, 1,4-benzoquinone, acetamide, oxalic acid, formic acid, 1,2,4-trihydroxybenzene, and maleic acid were the most frequent by-products of ACT.

**Table 3:** Number of detections of the most frequent by-products for different AOP systems

Carboxylic acids compounds and small by-products	Number of detections	Remark	Quinone derivatives and aromatic by-products	Number of detections	Remark	References
Acetamide	15	Acetamide one of the most frequent by products in all AOP systems	Hydroquinone	43	Is the most frequent by products in all AOP systems	15, 17, 31, 20, 32, 33, 72, 34, 30, 35, 76, 36, 29, 37, 38, 79, 80, 81, 82, 39, 83, 41, 40, 41, 84, 42, 85, 43, 86, 87, 44, 88, 89, 45, 46, 90, 69, 47, 91, 21, 92, 22, 93, 48, 49, 94, 50, 95–99, 27, 51, 52, 58, 53–55 and 100–103
Oxalic acid	14	Detected in all AOP systems except systems that based persulfate and peroxymonosulfate as an oxidant	1,4-Benzoquinone	26	Was detected after treatment of ATC by different AOP systems	
Formic acid	10	Detected in all AOP systems except systems that based irradiation as a catalyst	4-Aminophenol	16	Detected in all AOP systems except systems that based persulfate and peroxymonosulfate as an oxidant	
Acetic acid	9	Mostly detected after oxidation of ACT by electrooxidation systems	1,2,4-Trihydroxybenzene	5	This by-product was frequently detected after oxidation of ACT by photodegradation systems	
Oxamic acid	7	Detected in all AOP systems except systems that based persulfate and peroxymonosulfate as an oxidant	4-Methylbenzene-1,2-diol	2	Only observed after ACT treatment by systems that based persulfate and peroxymonosulfate as an oxidant	
Maleic acid	5	Only observed after ACT treatment by systems that photodegradation systems	Benzoic acid	2	Benzoic acid frequently detected after electrooxidation of ACT	
Malonic acid	2	Mostly detected after oxidation of ACT by electrooxidation systems	*N*-(3,4-Dihydroxyphenyl)formamide	2	Only observed after ACT treatment by systems that based persulfate and peroxymonosulfate as an oxidant	
4-Heptanol	2	Mostly detected after oxidation of ACT by electrooxidation systems				
Butanoic acid	2					
Hydroxyacetone	2					
2-(Acetylamino)-2-propenoic acid	2	Only observed after ACT treatment by systems that based persulfate and peroxymonosulfate as an oxidant				

## 5. The toxicity assessment of ACT and its by-products

The toxicity evaluation of ACT and its by-products is important to increase the system efficiency. It has been reported that by-products could be threatened and toxic for the environment and public health more than the parent pollutant itself. The toxicity assessment of ACT and its by-products were carried out by using the United States Environmental Protection Agency software called Toxicity Estimation Software Tool (TEST) version 5.1. This software is capable to apply mathematical models to predict pollutant toxicity based on Quantitative Structure Activity Relationship (QSAR) methodology. The data was introduced by inputting the name of each by-product. The Lethal concentration 50% (LC_50_) (96 h) fathead minnow and Ames mutagenicity were the considered toxicity text. The LC_50_ of prediction values for ACT was 813.76, and 123.08 mg L^−1^, respectively, and the mutagenicity test showing negative for both experimental and prediction tests. However, *N*-(3,4-dihydroxyphenyl) acetamide showed positive mutagenicity for both experimental and prediction tests. Meanwhile *N*-(2,4-dihydroxyphenyl) acetamide and malonic acid showed positive mutagenicity only for the prediction test. Table 4 represents the results of LC_50_ (96 h) fathead minnow and the mutagenicity tests for the most frequent by-product out of 64 studies collected in this work.

**Table 4:** Prediction values of (LC_50_) (96 h) fathead minnow and Ames mutagenicity for ACT by-products^a^

Name	Chemical structure	The percentage of the frequent by-products out of 64 studies				Fathead minnow, LC_50_ (96 h)		Ames mutagenicity	
		Photo catalytic%	Sulfate radical-AOP%	EO%	Other AOP process%	−log (mol L^−1^)	Predicted value (mg L^−1^)	Experimental result	Predicted result
**Toxic compounds**									
Benzaldehyde	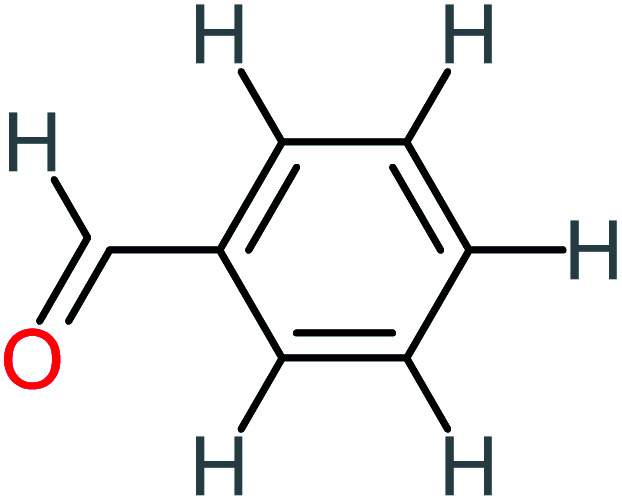	a	a	8%	8%	2.98	6.82	a	Mutagenicity negative

**Harmful compounds**									
Hydroquinone	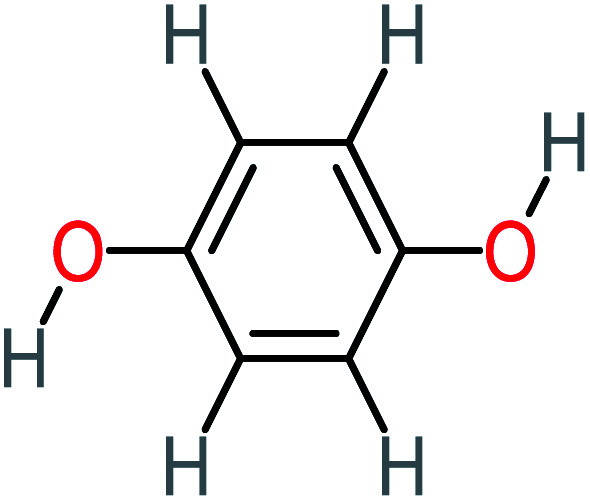	66%	67%	62%	58%	3.81	17.16	Mutagenicity negative	Mutagenicity negative
*N*-(3,4-Dihydroxyphenyl)formamide	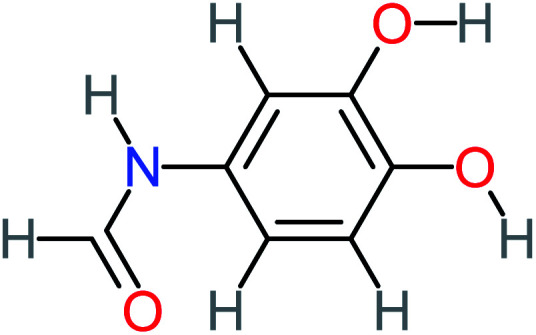	a	17%	a	a	3.52	45.86	a	Mutagenicity negative
4-Methylbenzene-1,2-diol	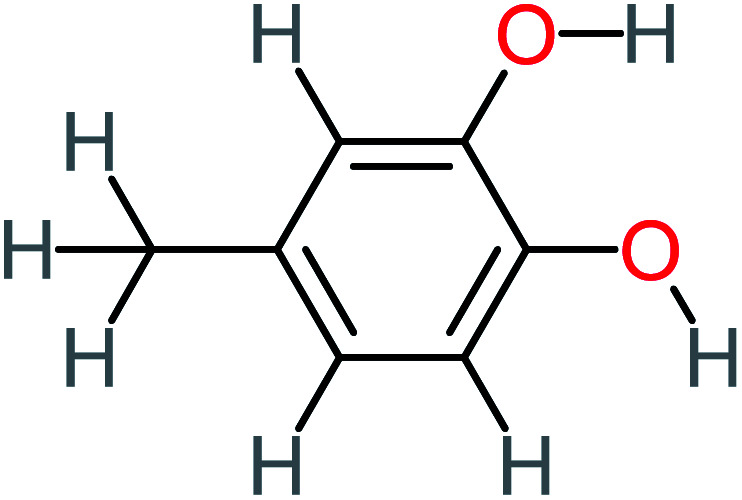	a	17%	a	a	3.65	27.57	Mutagenicity negative	Mutagenicity negative
Benzoquinone	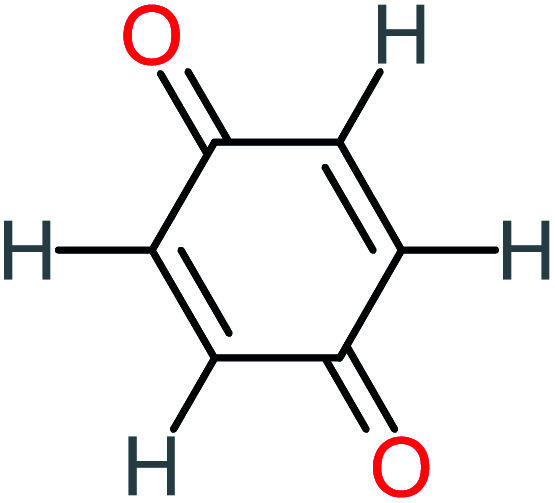	37%	33%	62%	33%	3.49	35.11	Mutagenicity negative	Mutagenicity negative
4-Aminophenol	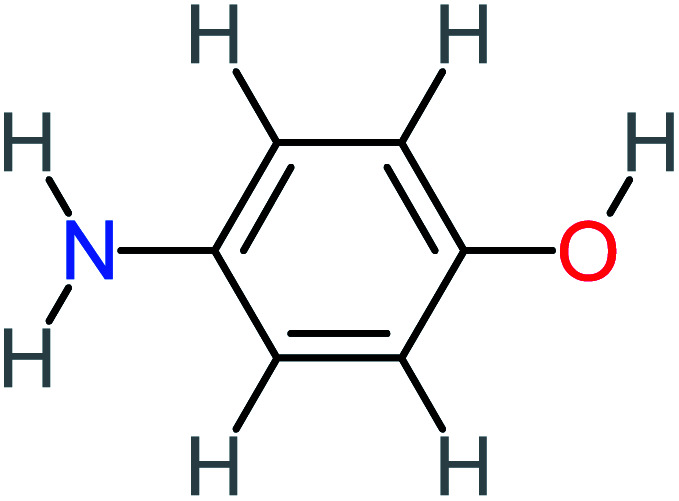	30%	42%	8%	25%	3.30	54.55	Mutagenicity negative	Mutagenicity negative
Benzoic acid	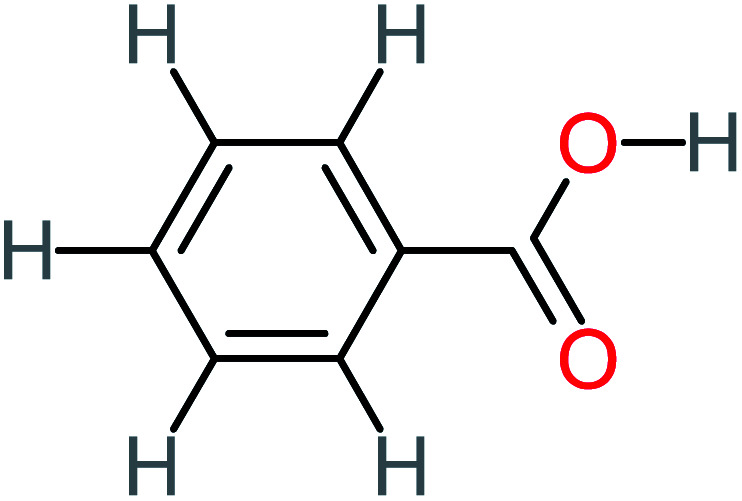	11%	a	15%	8%	3.21	75.43	Mutagenicity negative	Mutagenicity negative
1,2,4-Trihydroxybenzene	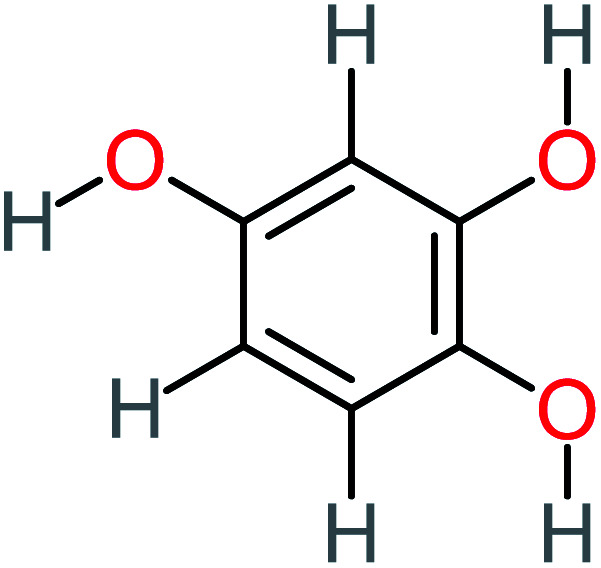	18%	8%	8%	a	3.01	24.64	Mutagenicity negative	Mutagenicity negative
4-Nitrophenol	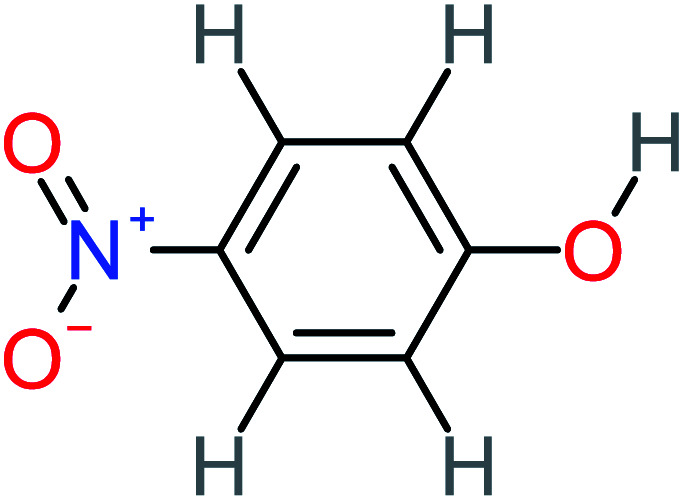	7%	25%	a	a	1.93	19.02	Mutagenicity negative	Mutagenicity negative
4-Aminobenzene-1,2-diol	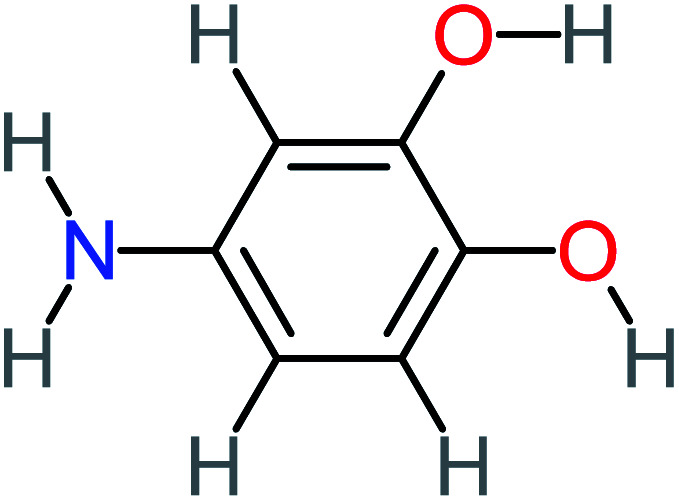	a	16%	a	a	3.86	43.18	Mutagenicity negative	Mutagenicity negative

**Harmless compounds**									
Hydroxy-acetic acid	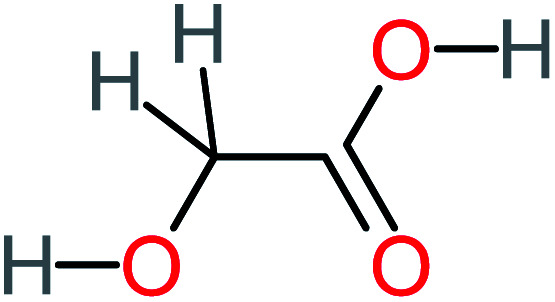	7%	a	8%	17%	1.60	1904.50	Mutagenicity negative	Mutagenicity negative
Malonic acid	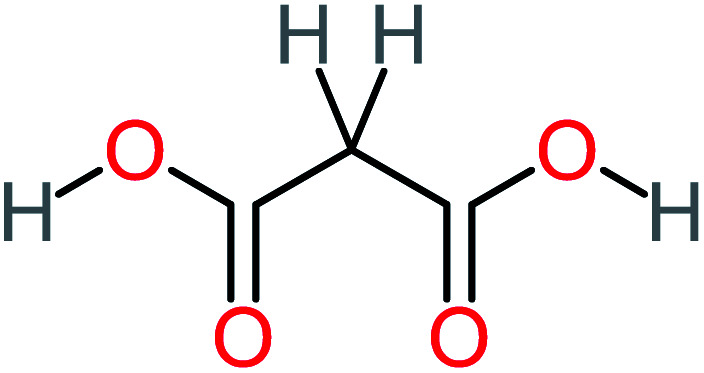	15%	8%	15%	8%	2.17	704.59	Mutagenicity negative	Mutagenicity positive
Succinic acid	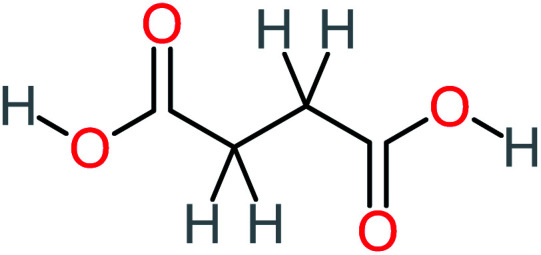	a	a	a	8%	2.51	367.61	a	Mutagenicity negative
Malic acid	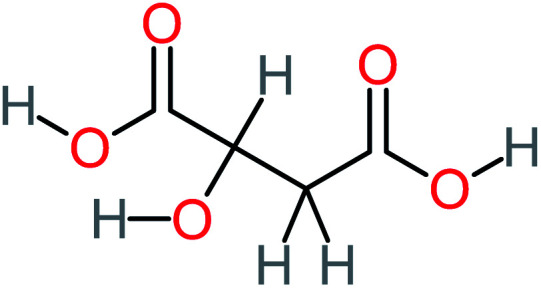	11%	a	8%	8%	2.40	529.53	a	Mutagenicity negative
Acetamide	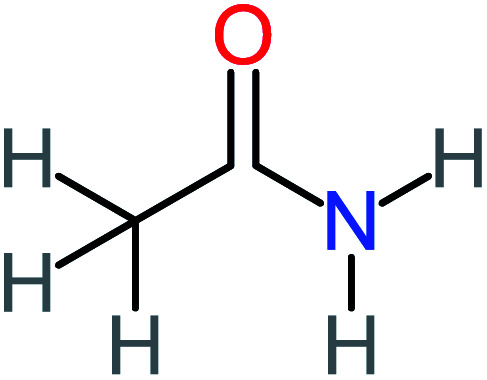	26%	8%	31%	33%	1.97	637.40	Mutagenicity negative	Mutagenicity negative
Tartronic acid	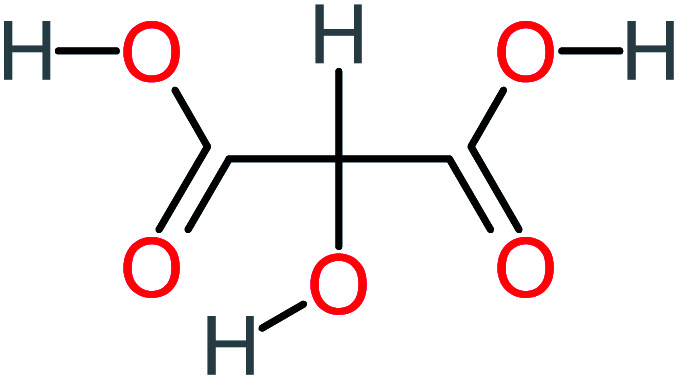	4%	a	a	8%	2.27	644.64	a	Mutagenicity negative
Maleic acid	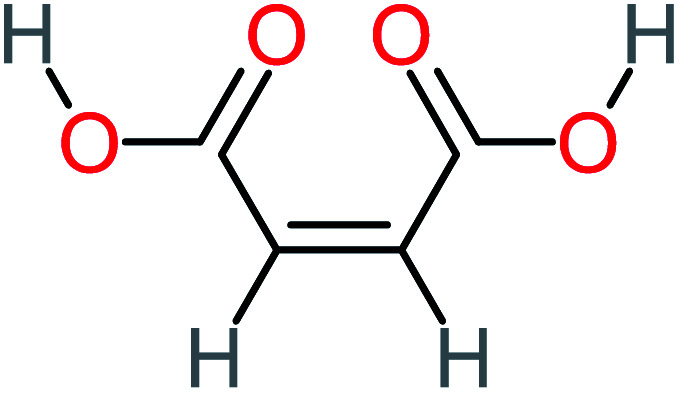	19%	a	8%	17%	2.59	298.69	Mutagenicity negative	Mutagenicity negative
Oxalic acid	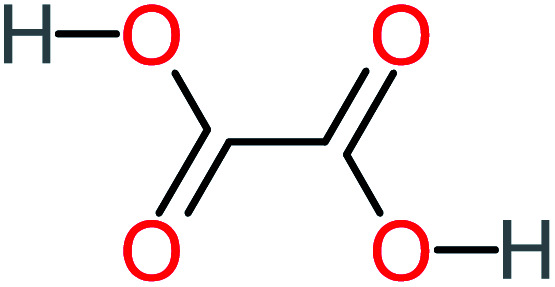	19%	a	38%	a	2.18	592.91	Mutagenicity negative	Mutagenicity negative
Oxamic acid	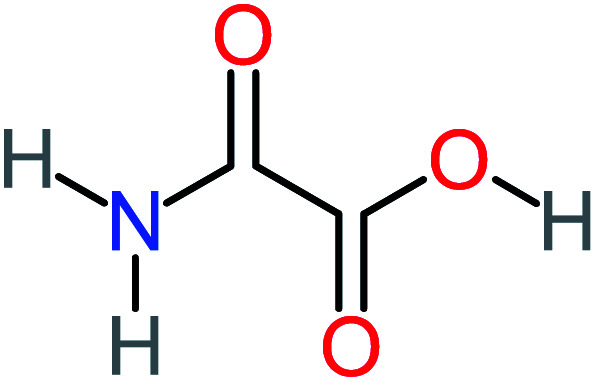	19%	a	15%	8%	2.23	520.93	a	Mutagenicity negative
Butyric acid	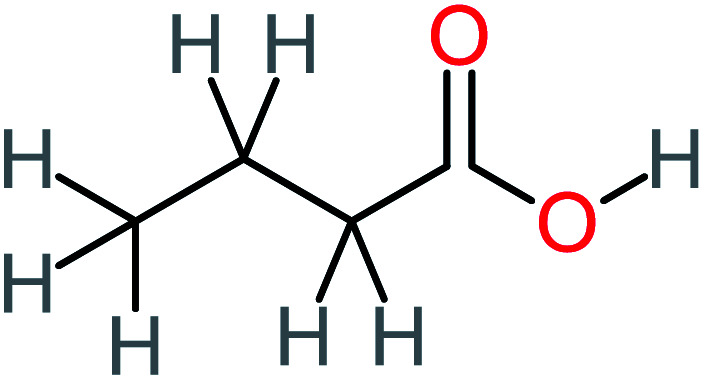	7%	a	a	a	2.29	448.12	Mutagenicity negative	Mutagenicity negative
Acetic acid	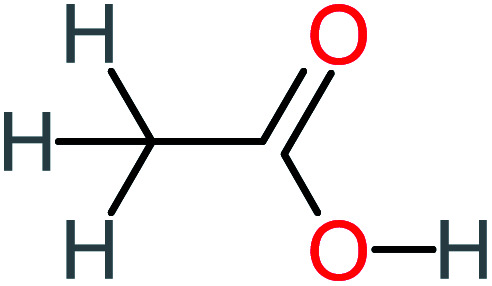	15%	a	23%	50%	2.66	132.49	Mutagenicity negative	Mutagenicity negative
*N*-(3,4-Dihydroxyphenyl) acetamide	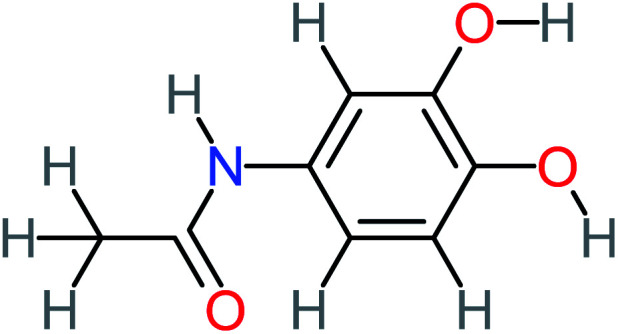	14%	8%	8%	a	3.71	125.65	Mutagenicity positive	Mutagenicity negative
4-Heptanol	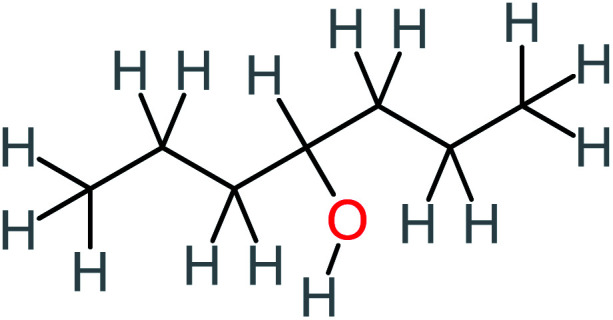	a	a	15%	a	a	122.16	a	a
Ethylamine	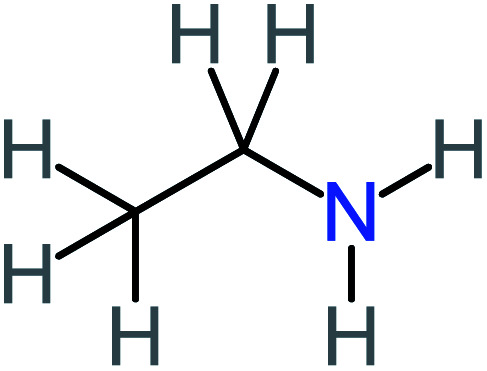	a	a	8%	8%	4.19	525.21	Mutagenicity negative	Mutagenicity negative
Hydroxyacetone	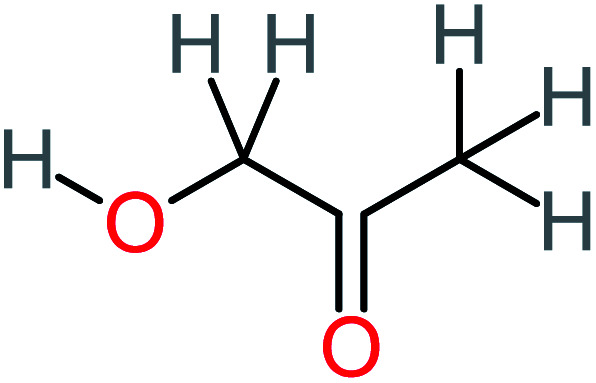	a	a	15%	a	a	3589.95	a	a
*N*-(2,4-Dihydroxyphenyl) acetamide	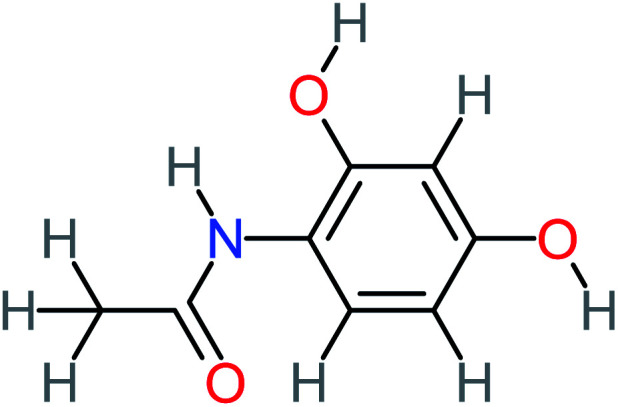	a	a	8%	8%	1.31	113.70	a	Mutagenicity positive

**Unknown toxicity values**									
ACT dimer	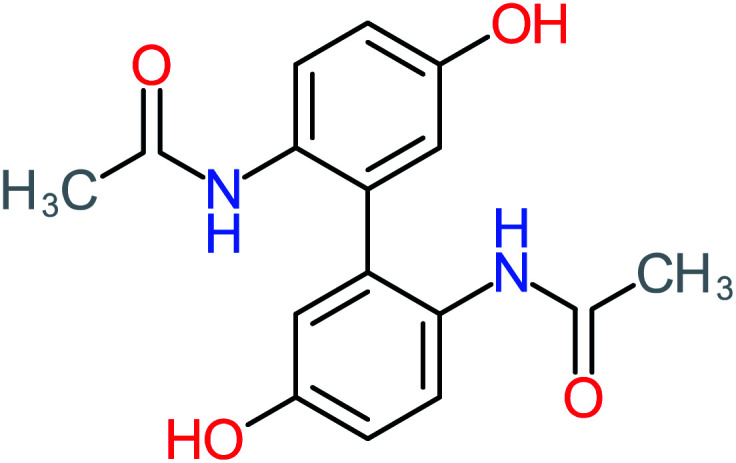	11%	a	a	8%	3.46	a	a	Mutagenicity negative
2-(Acetylamino)-2-propenoic acid	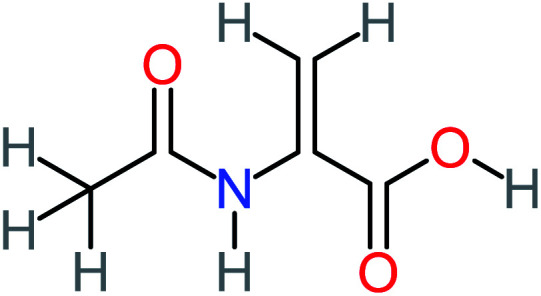	a	16%	a	a	a	a	a	a
2-Hydroxy-4-(*N*-acetyl) aminophenol	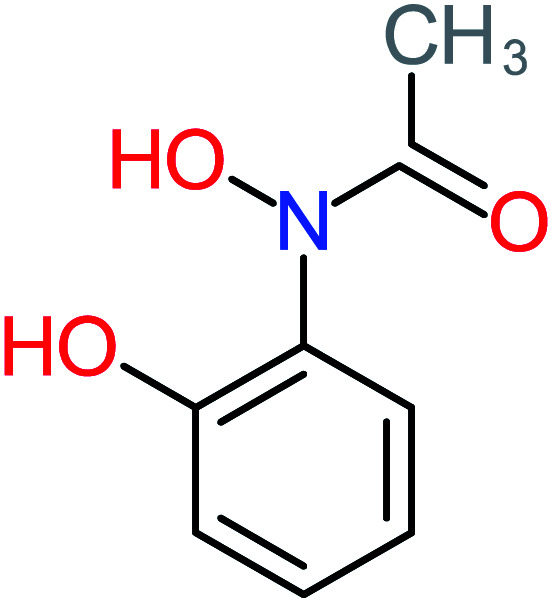	7%	a	a	a	a	a	a	a
Formic acid	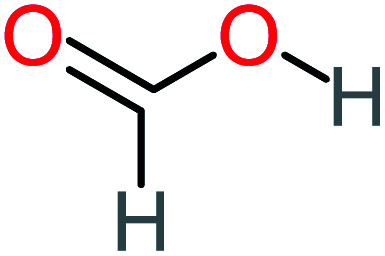	11%	17%	15%	50%	a	a	Mutagenicity negative	Mutagenicity negative

a Data not available.

The toxicity of all chemicals in the aquatic environment is classified into four categories according to the globally harmonized system GHS: extremely toxic, toxic, harmful, and harmless chemicals.^104^ The acute toxicity LC_50_ (96 h) for fathead minnow exposured to ACT by-products can classify harmless if the concentration of LC_50_ (96 h) within range from 1000 to 100 mg L^−1^, harmful level from 100 to 10 mg L^−1^, toxic level from 10 to 1 mg L^−1^, and very toxic level at values less than 1 mg L^−1^.^28^ The findings revealed that toxic reaction of hydroxy-acetic acid, malonic acid, succinic acid, malic acid, acetamide, tartronic acid, maleic acid, oxalic acid, oxamic acid, butyric acid, acetic acid, *N*-(3,4-dihydroxyphenyl)acetamide, 4-heptanol, ethylamine, hydroxyacetone, and *N*-(2,4-dihydroxyphenyl) acetamide to the fathead minnow organism was belonged to the harmless level. Besides, hydroquinone, *N*-(3,4-dihydroxyphenyl)formamide, 4-methylbenzene-1,2-diol, benzoquinone, 4-aminophenol, benzoic acid, 1,2,4-trihydroxybenzene, 4-nitrophenol, and 4-aminobenzene-1,2-diol belongs to harmful level. Furthermore, benzaldehyde is a toxic by-product for fathead minnow. Extremely toxic level of ACT by-product has not been detected in all AOP system that applied.

The following literature provides some experimental toxicity assessment of ACT and its frequent by-products such as hydroquinone, benzoquinone, benzaldehyde, and benzoic acid. For example, Nunes *et al.*^105^ examined the influence of acute exposure of ACT onto two aquatic plants *Lemna gibba* and *Lemna minor*. They revealed that ACT had a significant impact on the number of *Lemna minor* fronds (EC_50_ = 446.6 mg L^−1^), but there was no effect on *Lemna gibba*. Xu *et al.*^106^ studied the acute and chronicle effects of ACT onto three different aquatic species (i) fish, (ii) green algae and (iii) daphnia. The acute toxicity values were LC_50_ = 63.1 mg L^−1^ for daphnia and LC_50_ = 323 mg L^−1^ for fish, and EC_50_ = 26.3 mg L^−1^. The chronicle concentrations were 26.3, 5.13, 37.2 mg L^−1^, for fish, daphnia, and green algae, respectively. The author concluded that there was no adverse effect at chronical value for green algae and fish, but it was harmful to daphnia. Moreover, Sung *et al.*^107^ studied the acute toxicity of ACT on shrimp *Neocaridina denticulate*. The results revealed that the LC_50_ = 6.6 mg L^−1^ after 96 hours of exposure. Kataoka *et al.*^108^ proposed that the toxicity of ACT on aquatic organisms depends on environmental temperature. They used *Oryzias latipes* to examine their hypothesis because *Oryzias latipes* can live at a wide range of temperatures from 0 to 40 °C. The egg yolk of *Oryzias latipes* exposures to many ACT concentrations at different temperatures 15, 25, and 30 °C for 4 days. The authors revealed that, in any ACT concentrations, the absorption of ACT by egg yolk increased with increasing temperature. Based on the hematological analysis showed at 150 mg L^−1^ of ACT, the abnormal red cells were increased. In addition, previous researches showed that ACT negatively impacted zebrafish (*Danio rerio*). For example, Galus *et al.*^109^ studied the negative influences of different ACT concentrations from 0.05 μg L^−1^ to 50 μg L^−1^ on *Danio rerio*. The results indicated that at low ACT concentration 0.1 μg L^−1^, the abnormality was sharply increased, and all test concentrations showed increases in mortality rates. Erhunmwunse *et al.*^110^ investigated the acute effects of ACT on developmental, swimming performance, and cardiovascular activities on larvae (*Clarias gariepinus*). In this study, a fish embryo acute toxicity test was applied. Many ACT concentrations were exposed into *Clarias gariepinus* embryo 0, 0.5, 1, and 10 μg L^−1^, and the results concluded that ACT caused teratogenic, neurotoxic, and cardiotoxic effects into *Clarias gariepinus*.

Hydroquinone is widely used as a water-soluble constituent of foods, an antioxidant in industrial polymers, and as an ingredient in skin lightening preparations.^111^ The literature agreed that hydroquinone is a haematotoxin and carcinogenic agent, and well known its adverse effects on public health and the environment. A human might exposure to hydroquinone from many sources such as dietary, occupational, and environmental sources. O'Donoghue *et al.*^112^ studied the acute effect of hydroquinone on DNA damage *in vivo* comet assay in F344 rats. The results revealed that hydroquinone caused acute renal necrosis at dosage 420 mg per kg per day. Ji *et al.*^113^ examined cytogenetic changes in chromosomes 5, 7, 8, 11, and 21, and global DNA methylation in human TK6 lymphoblastoid cells were exposed for 48 houses with hydroquinone. In compared to melphalan and etoposide, the results revealed a worldwide hypomethylation at an intermediate level. They also discovered a cytogenetic change. Bährs *et al.*^114^ investigated the influence of pH and the time of hydroquinone exposure on the growth performance of different eukaryotic and prokaryotic freshwater phototrophs. The authors reported that cyanobacterial species were much more vulnerable to hydroquinone than coccal algal. The Microcystis aeruginosa species was the most sensitive by far. In addition, the impact of pH on hydroquinone toxicity was studied. At pH 11, the hydroquinone stock solution got polymerized, which led to the loss of its toxicity. On the other hand, the i potential was sustained if the polyphenol was kept at pH 7. Furthermore,^115^ studied the toxicity of hydroquinone on the white rabbit in New Zealand. Three different dosages were applied every day 0, 25, 75, and 150 mg per kg per day. The results revealed that 75 and 150 mg were negatively affected in the body weight and feed consumption during the experiment period. In addition,^116^ pointed out that hydroquinone was able to increase carcinogenic risk by generating DNA damage and compromising the general immune responses, which may contribute to the impaired triggering of the host immune reaction. They demonstrated that hydroquinone was more toxic for aquatic organisms than bacteria and fungi.^117^ studied the influence of multiple metabolites compounds such as 1,2,4-benzentriol, hydroquinone, 1,4-benzoquinone, 2,2-biphenol, and 4,4-biphenol on the DNA cleavage activity of human topoisomerase IIα. The results showed that hydroquinone and 1,4-bezoquinone were the most attributes against topoisomerase IIα, including DNA cleavage specificity. Hydroquinone also prevented DNA ligation more effectively than 1,4-benzoquinone.

According to the studies, 1,4-benzoquinone is a highly reactive metabolite that can be caused cells damages through forming DNA adducts and produce superoxide species. In addition, 1,4-benzoquinone can directly attack the macromolecules. Many adverse effects of benzoquinone have been investigated. For example,^118^ demonstrated that benzoquinone inhibited the cycle progression and induced the contraction and shrinkage of the A549 cells. Thus, leading to the direct effect of the damage of the microtubule cytoskeleton. Pengling Sun *et al.*^119^ examined the VNN3 gene code as a biomarker of the 1,4-benzoquinone toxicity. They cultured AHH-1 cells *in vitro* and incubated them with 0, 10, 20, and 40 mM of 1,4-benzoquinone for 24 hours. The results showed that 1,4-benzoquinone increases the expression of the VNN3 gene, thus leading to inhibit cell proliferation. Summary *et al.*^120^ studied the long and short term of exposure of quinone introduced *via* inhalation into human. They revealed that the acute exposure of quinone with high concentration resulted the following symptoms (i) consisting of discoloration of the conjunctiva and cornea (ii) causes dermatitis from dermal exposure (iii) irritation of the eyes. For long-term exposure appeared the following symptoms, causes skin ulceration and visual disturbances. Furthermore,^121^ reported that the ACT and 1,4-benzoquinone imine through intraperitoneal injections in the mouse. They mentioned that the LD_50_ values were 500 and 8.5 mg kg^−1^, for ACT and 1,4-benzoquinone. That means 1,4-benzoquinone higher 58 times than ACT.^122^ examined a new approach to determine the toxicity of 1,4-benzoquinone. The results revealed that the IC_50_ of 1,4-benzoquinone was 0.89 mg L^−1^, which means highly toxic, and its toxicity should not be ignored. Moreover, Faiola.^123^ revealed that 1,4-benzoquinone had a direct toxic effect in hematopoietic stem cells (HSCs), which rise to leukemic clones. Kondrová *et al.*^124^ studied the mechanisms of the oxidation stress of 1,4-benzoquinone on destroying cytochrome P450. The study observed that 1,4-benzoquinone mainly destroying cytochrome P450 by direct attack of the macromolecules.

Many studies including the chronic and acute effects of benzoic acid, benzaldehyde, and benzene derivatives on different organisms like humans, cats, rats, and other microorganisms. For example, Lee & Chen,^125^ studied the toxicity of benzoic acid and its derivatives on *Pseudokirchneriella subcapitata*. The results indicated that the EC_50_ range of benzoic acid was between 0.55 to 270.7 mg L^−1^. In addition, they revealed that benzene derivatives (2,4,6-trihydroxylbenzoic acid, 2,3,4-trihydroxylbenzoic acid, 2,6-dihydroxylbenzoic acid, 3-bromobenzoic acid, 4-bromobenzoic acid, and 4-chlorobenzoic acid, were more toxic than benzoic acid. In addition, Paulraj *et al.*^126^ examined the pupicidal and larvicidal, which are based on benzaldehyde applied on larvae and pupae stages of *Culex quinquefasciatus* and *Aedes aegypti*. They revealed that the LC_50_ of benzaldehyde on *Culex quinquefasciatus* and *Aedes aegypti* were 40.48 and 30.39 ppm after 12.08 and 9.44 min, respectively. The adult mortality of *Aedes aegypti* was reached 100% after 24 hours of treatment and the mortality of *Culex quinquefasciatus* was 100% by using in both benzaldehyde and propionic acid. Velegraki *et al.*^71^ investigated the influence of benzoic acid on sea bacteria *Vibrio fischeri* after the treatment process by an electrooxidation system. The results indicated that at initial concentration 50 mg L^−1^, of benzoic acid in the early stage of treatment was the most toxic with inhabitation around 80% of the bacteria after 6 hours of reaction, the inhibition was kept at 80%. After that, the inhibition started to decrease. Johnson *et al.*^127^ mentioned the acute inhalation exposure of benzoic acid for 4 hours introduced to a rat. The results indicated that low acute toxicity was observed on the rate. For oral dose toxicity, if the concentration of benzoic acid below is 800 mg per kg body weight per day, there were no observable adverse effects, while in the concentration of benzoic acid exceed 800 mg kg^−1^, there were adverse effects have appeared on the liver, kidney of the rat. Furthermore, Kreis *et al.*^128^ studied the toxicity of benzoic acid with high dosage and short-term exposure on rats, around 2250 mg kg^−1^ of benzoic acid was introduced into the rat within 5 days. The results showed around 50% of mortality and many critical adverse effects were observed on rats like histopathological alteration, ataxia, excitation, bleeding into the gut, and convulsion.

## 6. Future outlook

The development of AOPs as an effective approach to degrade ACT is more demanding of people's attention. The following are the main components of ACT treatment development using advanced oxidation technology:

• The degradation of ACT through radical and non-radical pathways can coexist in chemical oxidation. Since the radicals prefer to attack the more electrophilic sites on the pollutant, which can predict the degradation pathway through DFT method, while the non-radicle pathways attack the pollutant from any site spontaneously, which can generate a wide range of byproducts and increase the difficulty to apply DFT method.

• However, identifying the precise and quantitative contribution of radical and non-radical pathways in the overall oxidative response remains a difficulty, which reduce the preciseness of DFT method to predict the degradation pathways of target pollutant.

• Another important point to keep in mind is that most ACT degrading research has been done with simulated wastewater, with only a few studies concentrating on real wastewater. As a result, the presence of cations, anions, organic, and inorganic chemicals may act as an interference and may change the degradation pathway of ACT and their by-products.

## 7. Conclusions

This article has attempted to give a critical review for ACT by-products and their toxicity, proposed degradation pathways of ACT. In addition, the computational method was used to build the degradation pathways of ACT. The following point concludes the results of this study:

• This study revealed that the most of the by-products that frequently detected were hydroquinone, 1,4-benzoquinone, 4-aminophenol, acetamide, oxalic acid, formic acid, acetic acid, 1,2,4-trihydroxybenzene, and maleic acid, respectively.

• *N*-(3,4-Dihydroxyphenyl)acetamide showed positive mutagenicity for both experimental and prediction tests. Meanwhile, *N*-(2,4-dihydroxyphenyl)acetamide and malonic acid showed positive mutagenicity only for the prediction test. The findings of LC_50_ (96 h) test revealed that benzaldehyde is the most toxic ACT by-products and hydroquinone, *N*-(3,4-dihydroxyphenyl)formamide, 4-methylbenzene-1,2-diol, benzoquinone, 4-aminophenol, benzoic acid, 1,2,4-trihydroxybenzene, 4-nitrophenol, and 4-aminobenzene-1,2-diol considered harmful. The release of them into the environment without treatment may threaten the ecosystem.

• The degradation pathway of ACT based on the computational method was matched with the majority of ACT proposed pathways and matched with the most frequent ACT by-products.

## Conflicts of interest

There are no conflicts to declare.

## Supplementary Material
